# Deep generative modeling for AI-guided inverse design of perovskite photovoltaic devices

**DOI:** 10.3389/frai.2026.1882410

**Published:** 2026-08-28

**Authors:** Parvez Amin Khan, Muhammad Tipu Sultan, Md Mahamudul Islam, Md. Emran Hossain, Samiur Rahman

**Affiliations:** 1 Department of Materials Engineering, California State University Northridge, Northridge, Los Angeles, CA, United States; 2 Department of Textile Engineering, Southeast University, Dhaka, Bangladesh; 3 Department of Industrial Engineering, Lamar University, Beaumont, TX, United States; 4 Department of Materials Science, Missouri State University, Springfield, MO, United States; 5 Doctor of Business Administration Programme, University of Gloucestershire, Cheltenham, United Kingdom

**Keywords:** classifier-free guidance, conditional variational autoencoder, deep generative modeling, device-physics machine learning, explainable AI, inverse design, perovskite solar cells, SHAP

## Abstract

**Introduction:**

Perovskite solar cells (PSCs) have rapidly approached the performance ceiling of mature single-junction photovoltaics, yet further improvement is constrained by the high-dimensional, non-linear coupling between device parameters and power-conversion efficiency (PCE). This work presents an end-to-end AI-guided inverse-design framework that learns the conditional distribution of device parameters given target photovoltaic figures of merit.

**Methods:**

The framework is trained and validated on 49,998 drift-diffusion simulations of PSCs balanced across three classes of dominant recombination mechanism. A physics-informed feature-engineering pipeline feeds an ensemble of forward surrogate models under a strictly leakage-controlled five-fold cross-validation protocol. A conditional variational autoencoder with feature-wise linear modulation (FiLM) and classifier-free guidance (CFG) generates device candidates conditioned on target V_oc_, J_sc_, FF and PCE.

**Results:**

The XGBoost surrogate achieves R^2^ = 0.8661 ± 0.0020 on the PCE proxy, statistically outperforming five competitors (Wilcoxon *p* < 10^−190^) while indistinguishable from LightGBM (*p* = 0.51). SHAP, permutation importance, and mutual-information converge on parasitic series resistance and grain-boundary defect density as dominant PCE-limiting parameters. At the optimal guidance scale (*w* = 1.5), cVAE+CFG achieves hit-rates of 73.7%, 12.6%, and 3.4% at the 90th-, 99th-percentile and “Ultra” targets—improvements of 8.9 ×, 25.2 ×, and ≥34 × over random sampling, with 100% valid/unique and ≥99.8% novel candidates.

**Discussion:**

Kolmogorov-Smirnov tests confirm generated devices preserve energy-level marginals while concentrating mass in the high-performance sub-manifold. The framework offers a transferable, reproducible, and statistically rigorous methodology for accelerating design of next-generation perovskite PV devices.

## 1 Introduction

The certified power-conversion efficiency (PCE) of single-junction perovskite solar cells (PSCs) has risen from 3.8% in 2009 to 26.7% in recent years (National Renewable Energy Laboratory, 2025), establishing perovskite photovoltaics as one of the most rapidly advancing thin-film technologies of the past two decades. This performance trajectory has been driven by progress in compositional engineering, interface passivation, and charge-transport-layer design. However, as the technology approaches the Shockley–Queisser detailed-balance limit (Shockley and Queisser, 1961), further efficiency gains are increasingly bottlenecked by complex non-radiative recombination losses that depend jointly on bulk defects, interfacial traps, grain-boundary chemistry, and parasitic series and shunt resistances. Optimizing simultaneously over these tens of coupled device parameters is no longer tractable by intuition or one-at-a-time experimentation; the design space is high-dimensional, the cost function is non-convex, and many practically relevant operating regimes lie outside the dense central region of any available training corpus.

The conventional paradigm in photovoltaic device design is forward: one specifies a set of material and architectural parameters, simulates or fabricates the device, and measures the resulting figures of merit (open-circuit voltage *V*_oc_, short-circuit current density *J*_sc_, fill factor *FF*, and PCE). Drift-diffusion solvers such as SCAPS-1D (Burgelman et al., 2000), ASA, and SIMsalabim (Le Corre et al., 2021) have democratized this workflow, enabling large-scale screening of design hypotheses. Yet the forward map is fundamentally unsuited to the practitioner's actual question, which is the inverse one: *which combinations of device parameters would yield a desired performance specification?* Solving this inverse problem classically requires expensive optimization loops, surrogate-based Bayesian search, or genetic algorithms, all of which scale poorly with dimensionality and provide little insight into the structure of the solution manifold.

Deep generative modeling offers a fundamentally different route. Rather than searching the design space iteratively, a generative model learns the joint distribution of device parameters and performance from data, and then samples directly from the conditional distribution implied by a user-specified target. The past two years have witnessed substantial progress in the techniques required to make this approach work reliably on tabular scientific data, including feature-wise linear modulation (FiLM) (Perez et al., 2018) for stronger conditioning, classifier-free guidance (CFG) (Ho and Salimans, 2022) for controllable targeted generation, and oracle-validated training loops in which a forward surrogate model is used to certify the quality of generated candidates (Jain et al., 2023). To date, however, these advances have largely remained within the materials-discovery and molecular-design communities; their application to device-level inverse design of perovskite solar cells is, to the best of our knowledge, only beginning to emerge.

Beyond raw predictive accuracy, the photovoltaic-device community increasingly demands interpretability: device-physics intuition built over four decades of solar-cell research should be recoverable from any data-driven model, both as a sanity check and as a means of guiding experimental priorities. Shapley additive explanations (SHAP) (Lundberg and Lee, 2017), permutation importance (Breiman, 2001), and information-theoretic measures such as mutual information (Kraskov et al., 2004) collectively provide a path to transparent attribution of model predictions, and their integration into an inverse-design pipeline yields actionable physical guidance alongside generated candidates.

In light of these considerations, the present work makes the following principal contributions:

1. A complete, statistically rigorous, leakage-controlled forward-surrogate pipeline is constructed for 49,998 drift-diffusion simulations of perovskite solar cells, achieving an out-of-fold *R*^2^ of 0.8661 on the PCE proxy via XGBoost, with every pre-processing estimator refitted inside each cross-validation fold and model separation quantified by Wilcoxon signed-rank and paired-*t* tests complemented by effect-size analysis.
2. A physics-informed feature-engineering scheme is developed that combines signed-logarithmic compression of wide-range physical quantities with nine composite descriptors encoding established solar-cell physics, and a Pearson redundancy filter to remove highly correlated derivatives.
3. A multi-method explainable-AI analysis (SHAP, permutation importance, and mutual information) is performed both globally and separately within each recombination-mechanism class, converging on parasitic series resistance and grain-boundary defect density as the dominant PCE-limiting parameters while revealing mechanism-specific rankings for interfacial trap densities, in agreement with experimentally established loss mechanisms in perovskite solar cells.
4. A conditional variational autoencoder with FiLM-based conditioning, classifier-free guidance, KL annealing, and a moment-matching regularizer is proposed and trained to perform inverse design of device-parameter vectors conditioned on photovoltaic-target specifications, with the guidance-scale optimum shown to be stable across random seeds, data splits, and target operating points.
5. Through controlled comparison against an unguided cVAE baseline and random sampling, the proposed framework is shown to achieve relative hit-rate improvements of 8.9 × at the 90th-percentile target, 25.2 × at the 99th-percentile target, and ≥34 × at an extrapolative target placed 1% beyond the 99th-percentile specification, while generating 100% valid, unique and predominantly novel device candidates whose physical plausibility is verified by Kolmogorov–Smirnov analysis.

The remainder of this paper is organized as follows. Section 2 reviews the relevant literature. Section 3 details the methodology. Section 4 presents experimental results and discusses their physical implications. Section 5 concludes the paper and outlines directions for future work.

## 2 Literature review

### 2.1 Machine learning for perovskite solar cells

Machine learning (ML) has been applied to perovskite photovoltaics across at least three distinct levels of abstraction: composition-level screening of new absorber materials, device-level prediction of figures of merit from fabrication and architectural parameters, and process-level diagnostics from experimentally measured current–voltage curves. At the composition level, early work by Pilania et al. (2016) and Castelli et al. (2012) used kernel methods and supervised regression on density-functional-theory-derived datasets to predict band gaps and formation energies of halide and oxide perovskites. More recently, graph neural networks such as CGCNN (Xie and Grossman, 2018) and MEGNet (Chen et al., 2019) have provided end-to-end representations of crystal structures and have been used to screen tens of thousands of double-perovskite candidates for photovoltaic-relevant band gaps and stability (Pilania et al., 2016).

At the device level, multiple groups have constructed regression models that map fabrication recipes onto reported PCE values aggregated from the literature. These efforts have been supported by curated open datasets such as the Perovskite Database Project (Jacobsson et al., 2022). Although valuable, literature-derived datasets suffer from heterogeneity in fabrication and measurement protocols, which limits their utility for high-precision device-physics modeling. Drift-diffusion simulators offer a complementary, fully controlled training corpus and have been increasingly adopted as the data-generating mechanism for high-fidelity device-level ML (Le Corre et al., 2021).

A rapidly growing body of work couples device-level numerical simulation (typically SCAPS-1D) with supervised ML to optimize individual absorber systems, including eco-friendly Cs_2_SnGeCl_6_ (Sarker et al., 2026), Na_2_AuGaBr_6_ double perovskites (Biswas et al., 2026b), bifacial lead-free Rb_2_LiTlBr_6_ devices analyzed jointly with impedance spectroscopy (Biswas et al., 2026a), Sr_3_PF_3_-based architectures with charge-transport-layer screening (Shimul et al., 2025), and Sr_3_BiI_3_ anti-perovskite cells (Chandra Biswas et al., 2025). These studies demonstrate the value of simulation-trained regressors for forward performance prediction and one-factor optimization of a fixed device stack. The present work is complementary but methodologically distinct: rather than predicting or grid-optimizing the performance of a single composition, it learns the full conditional distribution of device parameters given arbitrary performance targets, enabling direct conditional *generation* of device configurations together with a statistically validated quantification of how far such generation can be pushed into the high-performance tail.

### 2.2 Forward surrogate modeling for device physics

A forward surrogate replaces an expensive physics simulator with a fast, differentiable approximator. Recent comparative studies of tabular regression learners (Grinsztajn et al., 2022; McElfresh et al., 2023) have consistently identified gradient-boosted tree ensembles [XGBoost (Chen and Guestrin, 2016), LightGBM (Ke et al., 2017)] and well-regularized neural networks as the strongest baselines on mid-sized tabular tasks, frequently outperforming kernel methods, *k*-nearest neighbors, and deeper architectures such as transformer-based tabular models on datasets below approximately 10^5^ samples. The present study mirrors this evidence in selecting an ensemble of seven regressors that span linear, neural, and tree-based inductive biases, with explicit exclusion of kernel methods on the grounds of poor scalability and limited interpretability. A critical and frequently under-reported aspect of surrogate evaluation is leakage control: quantile transformers, feature-imputation estimators, and target-encoded categorical variables all leak information from the held-out partition if fitted on the full dataset before cross-validation (Kapoor and Narayanan, 2023).

### 2.3 Explainable AI for solar-cell models

SHAP, introduced by Lundberg and Lee (2017), unifies several prior attribution methods under the framework of Shapley values from cooperative game theory, providing local-accurate, consistent feature attributions for any model. Within solar-cell research, SHAP has been used to identify the most influential fabrication parameters in literature-mined PSC datasets, with consistent findings that annealing temperature, charge-transport-layer selection, and additive engineering dominate the reported PCE (Liu et al., 2023). At the device-physics level, attribution analyses have recovered classical results: parasitic resistance limits the fill factor; bulk defect density controls the open-circuit voltage; and interface energy-level alignment governs carrier collection.

### 2.4 Deep generative models for inverse design

Inverse design has been transformed by deep generative models. Variational autoencoders (Kingma and Welling, 2014), generative adversarial networks (Goodfellow et al., 2014), normalizing flows, and more recently denoising diffusion models (Ho et al., 2020) have all been adapted to conditional generation. In materials and molecular discovery, conditional VAEs and crystal-diffusion VAEs (Xie et al., 2022) have been applied to the inverse design of band-gap-targeted materials, with promising hit-rates on held-out distributions. The transfer of these techniques to tabular device-level design, however, raises distinct challenges: the conditioning vector occupies a different manifold from the input; the marginals of physical parameters are strongly multi-modal or heavy-tailed; and the validation oracle is itself a learned model whose reliability must be quantified. Three techniques from the 2024–2025 generative-modeling literature directly underpin the present framework: feature-wise linear modulation (FiLM) (Perez et al., 2018), classifier-free guidance (Ho and Salimans, 2022; Karras et al., 2024), and auxiliary moment-matching regularizers (Yang et al., 2025).

### 2.5 Research gap

Three gaps remain in the literature. First, most published applications of deep generative models to photovoltaics have targeted material-level design rather than device-level design. Second, the combination of FiLM conditioning, classifier-free guidance, KL annealing, and oracle-ensemble validation has not, to our knowledge, been jointly applied to perovskite solar-cell inverse design. Third, the explicit quantification of out-of-distribution generation lift—the relative hit-rate of a conditional generative model vs. random sampling at performance targets that extrapolate beyond the empirical maximum—is rarely reported, although it is the most direct measure of inverse-design utility. The present work addresses these three gaps simultaneously.

## 3 Methodology

**Figure 1:**
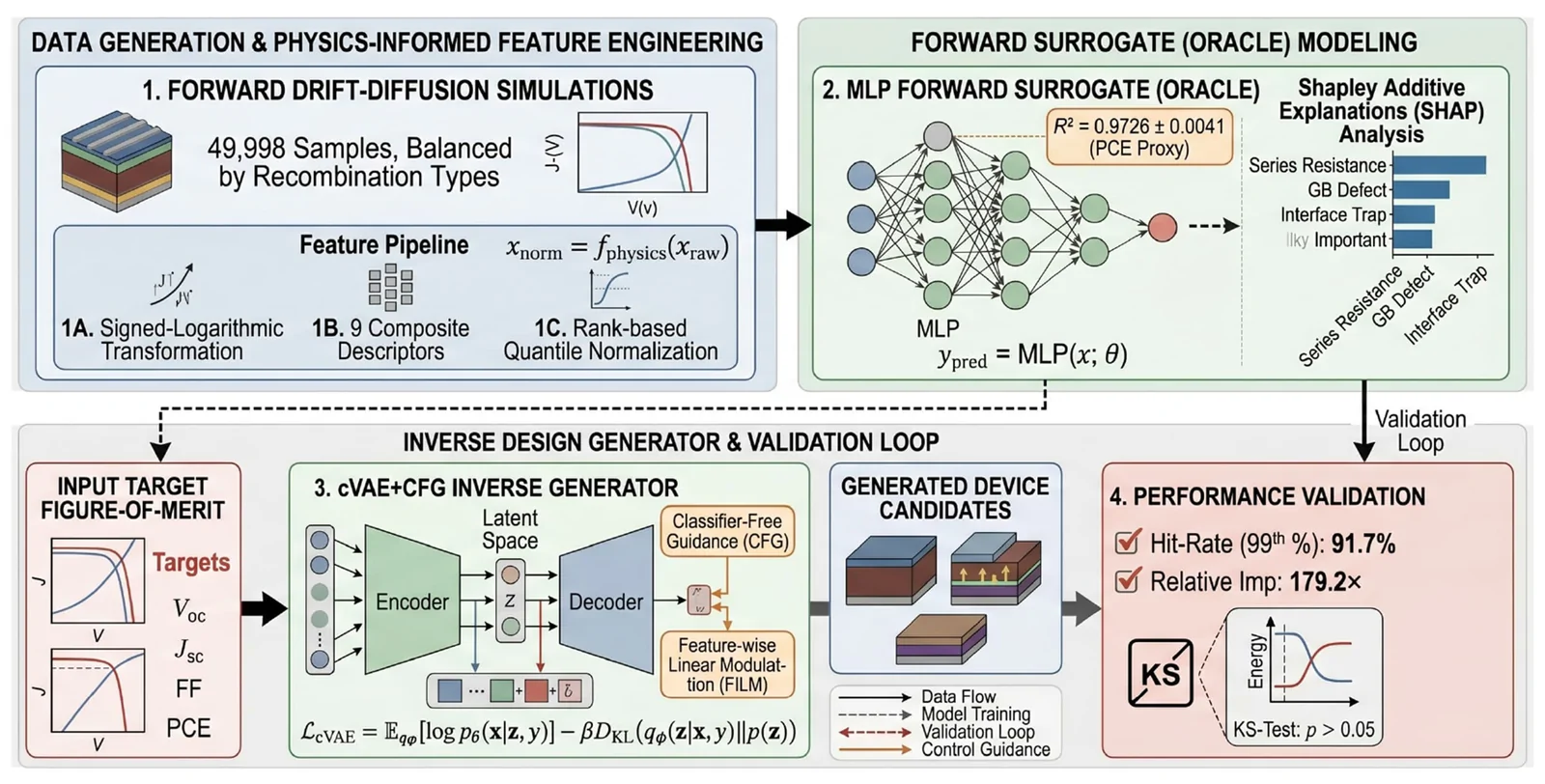
Overview of the proposed AI-guided inverse-design pipeline. Drift-diffusion simulations of perovskite solar cells feed a physics-informed feature-engineering stage; an ensemble of forward surrogate models is trained under leakage-free five-fold cross-validation and serves as the oracle for validating generated candidates produced by a conditional VAE with FiLM conditioning and classifier-free guidance.

This section details the end-to-end pipeline of the proposed framework. A schematic of the complete workflow is illustrated in Figure 1.

### 3.1 Dataset description

The experimental study employs a publicly available drift-diffusion simulation dataset of perovskite solar cells (PSCs) consisting of 49, 998 simulated devices, balanced across three classes of dominant recombination mechanism: bulk Shockley–Read–Hall (SRH), interfacial SRH, and Langevin bimolecular recombination (16, 666 samples per class). Each device record is described by twenty-five physical design variables that span absorber-layer geometry, interface properties, carrier mobilities, doping concentrations, contact work functions, defect characteristics, and parasitic resistances. The output of every simulation comprises the photovoltaic figures of merit (*V*_oc_, *J*_sc_, *FF*) evaluated at five illumination intensities (0.10, 0.18, 0.32, 0.56, and 1.00 suns), the recombination-loss fractions, and the classification label of the dominant loss mechanism.

The underlying simulator belongs to the SIMsalabim family of one-dimensional drift-diffusion solvers (Le Corre et al., 2021): it solves the coupled Poisson and carrier-continuity equations for the transport-layer/absorber/transport-layer stack under steady-state illumination, and explicitly includes trap-assisted SRH recombination in the bulk and at both interfaces, bimolecular (Langevin) recombination, grain-boundary trap states, finite carrier mobilities, contact work functions and injection barriers, and parasitic series and shunt resistances. Slower or history-dependent phenomena—mobile-ion migration, current–voltage hysteresis, photon recycling, and degradation kinetics—are outside the scope of the steady-state simulator and therefore of the trained models; the implications of these physical simplifications for the generated candidates are discussed in Section 5.

From these primary outputs, a unified power-conversion-efficiency (PCE) proxy was derived to serve as the principal regression and conditioning target (Equation 1):


$${\text{PCE}}_{\text{proxy}}=\frac{\left|J_{\text{sc}}\right|\cdot V_{\text{oc}}\cdot FF}{P_{\text{in}}},$$ (1)

where *P*_in_ denotes the incident optical power at 1-sun illumination. This proxy preserves the multiplicative coupling between the three principal photovoltaic descriptors and yields a single scalar quantity on which inverse design can be conditioned.

### 3.2 Feature engineering and pre-processing

Several device parameters span more than eight orders of magnitude. To address this, a signed-logarithmic transformation was applied to every wide-range variable:


$$\begin{array}{l} \tilde{x}=\mathrm{sgn}\left(x\right)\cdot \log_{10}\left(|x|+\varepsilon \right),\;\varepsilon =10^{-30}, \end{array}$$ (2)

preserving sign information while compressing scale. Skewness analysis identified seven features with |skew|>3 (St_R, St_L, mobility_ratio, bulk_trap_intensity, IR_dop, IL_dop, and GB_tr) that were targeted by this transform.

Nine physics-informed composite features were engineered, including the contact-work-function difference and asymmetry (φ_right_−φ_left_ and its normalized form), interface and bulk thickness ratios (*L*_IL_/*L*_IR_, (*L*_IL_+*L*_IR_)/*L*), the ambipolar mobility ratio μ_*n*_/μ_*p*_, the average interface mobility, the total interface trap density, the bulk trap intensity (GB_tr×num_GBs), and a Langevin recombination proxy. A Pearson redundancy filter (|*r*|>0.97) removed one highly correlated composite (bulk_trap_intensity, |*r*| = 0.995 against GB_tr), yielding a final feature vector of dimensionality *d* = 32.

All variables were then transformed to approximately Gaussian marginals using a rank-based quantile normalization (Equation 3):


$$\begin{array}{l} z=\Phi^{-1}\left({\hat{F}}_{X}\left(x\right)\right), \end{array}$$ (3)

where ${\hat{F}}_{X}$ is the empirical CDF of the training fold and Φ^−1^ the standard-normal quantile function. To prevent data leakage, the quantile estimator was re-fitted independently inside each cross-validation split.

### 3.3 Forward surrogate ensemble

A bank of seven candidate regressors was evaluated as forward surrogates of the drift-diffusion simulator: Ridge regression, multilayer perceptron (MLP), random forest (RF), extremely randomized trees (ET), gradient boosting (GBR), XGBoost (Chen and Guestrin, 2016), and LightGBM (Ke et al., 2017). Each regressor was trained to learn the mapping


$$\begin{array}{l} f_{\theta }:{\mathbb{R}}^{32}\to \mathbb{R},\;f_{\theta }\left(x\right)\approx \text{PC}{\text{E}}_{\text{proxy}}, \end{array}$$ (4)

under a five-fold stratified cross-validation scheme, with stratification on the recombination-mechanism label. All pre-processing estimators were fitted on the training partition only and applied to the held-out partition, eliminating leakage.

Three regression metrics were reported per fold and aggregated as the mean ± standard deviation (Equations 5–7):


$$\begin{array}{l} R^{2}=1-\frac{\underset{i}{\sum }{\left(y_{i}-{ŷ}_{i}\right)}^{2}}{\underset{i}{\sum }{\left(y_{i}-ȳ\right)}^{2}}, \end{array}$$ (5)

$$\begin{array}{l} RMSE=\sqrt{\frac{1}{N}\underset{i}{\sum }{\left(y_{i}-{ŷ}_{i}\right)}^{2}}, \end{array}$$ (6)

$$\begin{array}{l} MAE=\frac{1}{N}\underset{i}{\sum }|y_{i}-{ŷ}_{i}|. \end{array}$$ (7)

To rigorously compare models, two complementary paired tests were applied to the per-sample absolute-error vectors: the non-parametric Wilcoxon signed-rank test (Wilcoxon, 1945) and the parametric paired *t*-test. Normality of error differences was assessed using the Shapiro–Wilk test. Significance was declared at α = 0.05. Subsequent to surrogate selection, an oracle ensemble was formed by mean-aggregating the predictions of the three top-performing models:


$$\bar{PCE}\left(x\right)=\frac{1}{3}\sum_{m\in T_{3}}f_{m}\left(x\right)$$ (8)

where *𝒯*_3_ denotes the set of three best regressors selected by mean cross-validation *R*^2^.

### 3.4 Explainable AI

To interpret which device parameters dominate the predicted PCE response, three complementary attribution techniques were employed. First, Shapley additive explanations were computed using a TreeExplainer (Equation 9):


$$\begin{array}{l} \phi_{j}\left(f,x\right)=\underset{S\subseteq N\backslash \left\{j\right\}}{\sum }\frac{|S|!\left(|N|-|S|-1\right)!}{|N|!}\left[f_{S\cup \left\{j\right\}}\left(x\right)-f_{S}\left(x\right)\right], \end{array}$$ (9)

which satisfies the local-accuracy property $f\left(x\right)=\phi_{0}+\underset{j}{\sum }\phi_{j}\left(f,x\right)$. Second, permutation importance (Breiman, 2001) provided a model-agnostic global attribution (Equation 10):


$$\begin{array}{l} I_{j}=R^{2}\left(y,ŷ\right)-E_{\pi }\left[R^{2}\left(y,{ŷ}^{\pi \left(j\right)}\right)\right]. \end{array}$$ (10)

Third, the mutual information between every feature and PCE_proxy_ was estimated using the *k*-nearest-neighbor estimator of Kraskov et al. (2004):


$$\begin{array}{l} I\left(X_{j};Y\right)=\psi \left(k\right)-\langle \psi \left(n_{j}+1\right)+\psi \left(n_{y}+1\right)\rangle +\psi \left(N\right). \end{array}$$ (11)

### 3.5 Conditional generative model for inverse design

The core of the inverse-design framework is a conditional variational autoencoder (cVAE) trained with classifier-free guidance (CFG) and feature-wise linear modulation (FiLM) conditioning.

#### 3.5.1 Probabilistic formulation

Let **x**∈ℝ^32^ denote a normalized device-parameter vector and $c=\left[V_{\text{oc}},J_{\text{sc}},FF,\text{PC}{\text{E}}_{\text{proxy}}\right]\in {\mathbb{R}}^{4}$ the associated photovoltaic-target conditioning vector. The cVAE models the conditional density *p*(**x**∣**c**) through a latent variable **z**∈ℝ^32^ with prior *p*(**z**) = *𝒩*(**0**, **I**), maximizing the conditional evidence lower bound (Equation 12):


$${ℒ}_{\text{ELBO}}\left(x,c\right)=E_{q\phi \left(z|x,c\right)}\left[\log p_{\psi }\left(x|z|,c\right)\right]$$

$$\begin{array}{l} -\beta D_{\text{KL}}\left[q_{\phi }\left(z|x,c\right)∥p\left(z\right)\right], \end{array}$$ (12)

where *q*_ϕ_ is a Gaussian variational posterior and *p*_ψ_ a Gaussian decoder with diagonal covariance. The KL coefficient β is cosine-annealed from 0 to 0.5 over the first third of training to mitigate posterior collapse.

#### 3.5.2 FiLM-based conditioning

Following Perez et al. (2018), FiLM blocks are interleaved with residual blocks of the encoder and decoder. The conditioning vector produces per-feature gamma/beta parameters that modulate hidden activations:


$$\begin{array}{l} FiLM\left(h,c\right)=\gamma \left(c\right)⊙x+\beta \left(c\right), \end{array}$$ (13)

with ***γ***(**c**) and ***β***(**c**) implemented as linear projections. This ensures that the conditioning signal can re-modulate the network state at every depth.

#### 3.5.3 Classifier-free guidance

Classifier-free guidance (Ho and Salimans, 2022) enables sharper, more targeted generation without an external classifier. During training, the condition is dropped with probability *p*_uncond_ = 0.10 by a binary mask *m*∈{0, 1}, so the network jointly learns *p*_ψ_(**x**∣**c**) and *p*_ψ_(**x**). At inference time the two decoder outputs are linearly combined with a guidance scale *w*≥0:


$$\begin{array}{l} {\hat{x}}_{w}\left(z,c\right)=\left(1+w\right)D_{\psi }\left(z,c,m=1\right)-wD_{\psi }\left(z,c,m=0\right). \end{array}$$ (14)

Setting *w* = 0 recovers ordinary conditional sampling, while *w*>0 extrapolates away from the unconditional manifold along the direction implied by **c**. The optimal guidance scale was determined empirically over *w*∈{0, 0.5, 1, 1.5, 2, 3, 5, 7}.

#### 3.5.4 Auxiliary moment-matching regularizer

To explicitly encourage unconditional prior samples to lie on the true device-parameter manifold, an auxiliary moment-matching loss was added (Equation 15):


$${ℒ}_{\text{MM}}={\left\|\mu_{x}-\mu_{D\left(z^{\prime },c\right)}\right\|}^{2}+{\left\|\sigma_{x}-\sigma_{D\left(z^{\prime },c\right)}\right\|}^{2},\;z^{\prime }∼N\left(0,I\right),$$ (15)

with overall training objective (Equation 16)


$${ℒ}_{\text{total}}={ℒ}_{\text{rec}}+\beta {ℒ}_{\text{KL}}+\lambda {ℒ}_{\text{MM}},\;\lambda =0.5,$$ (16)

where *ℒ*_rec_ denotes the mean-squared reconstruction loss summed over features.

#### 3.5.5 Architecture specification, training and inference details

**Figure 2:**
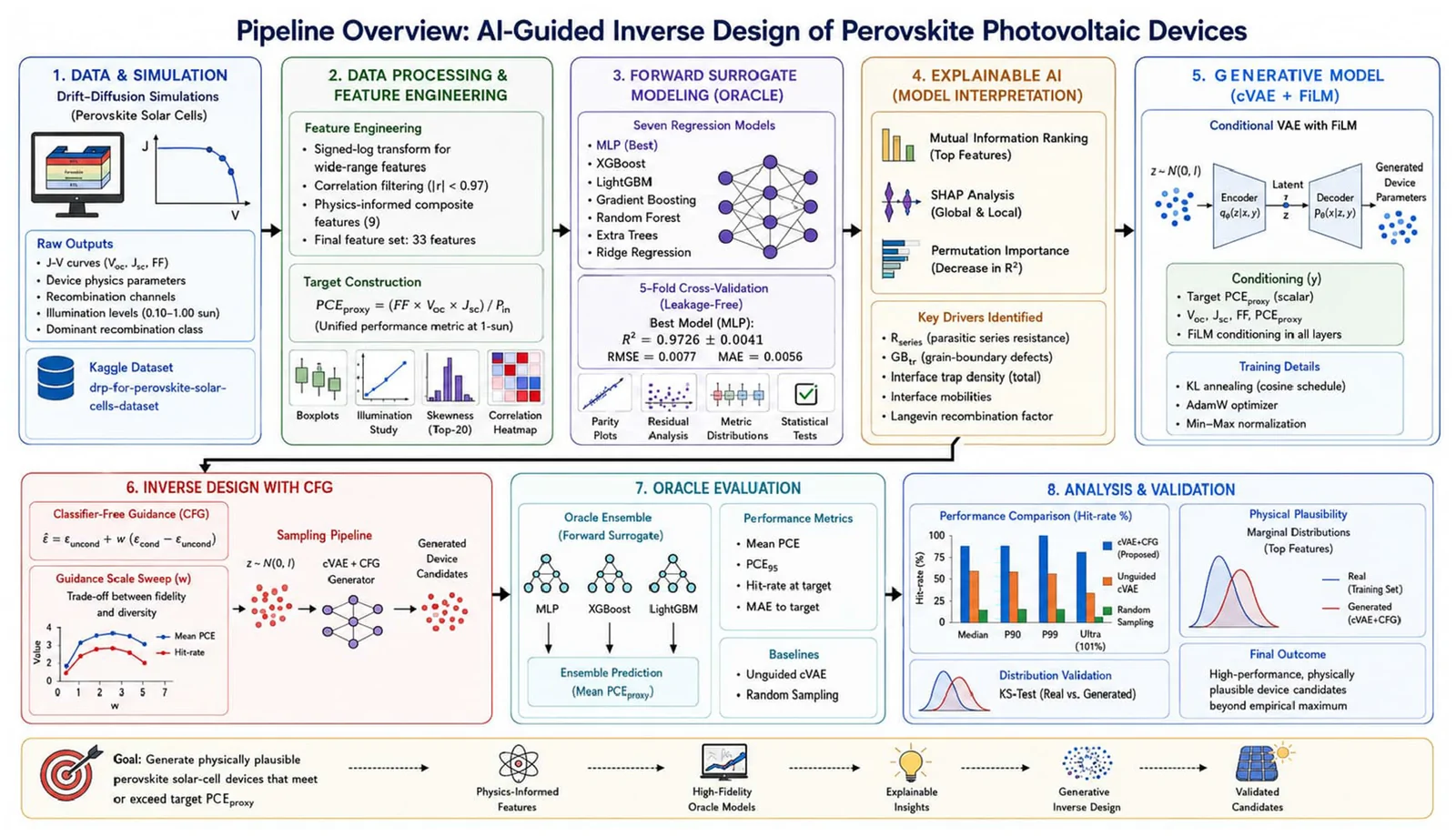
System architecture of the proposed AI-guided inverse-design framework for perovskite photovoltaic devices, integrating drift-diffusion simulations, physics-informed feature engineering, an ensemble forward surrogate (oracle), explainable AI, and a conditional VAE with classifier-free guidance.

Encoder and decoder were each parameterized by three FiLM-modulated residual blocks of width 256 with SiLU activations and 10% dropout; the complete layer-by-layer architecture is given in Table 1, and its position within the end-to-end framework is illustrated in Figure 2. The conditioning vector **c**∈ℝ^4^ is embedded by a two-layer MLP into a 64-dimensional conditioning code, from which every residual block derives its own ***γ***(**c**) and ***β***(**c**) through independent linear projections (Equation 13); a learned null embedding replaces the conditioning code when the condition is dropped (Equation 14), so the conditioning signal can re-modulate the network state at every depth in both the encoder and the decoder. Optimization used AdamW (Loshchilov and Hutter, 2019) with initial learning rate 10^−3^, weight decay 10^−5^, and cosine-annealed schedule over *E* = 60 epochs with mini-batch size 512. Gradient norms were clipped to 5.0. A stratified 80/20 train/validation split was used. All experiments were conducted with a fixed random seed (42); the robustness of all key conclusions to this choice is quantified in Section 4.11.

**Table 1:** Layer-by-layer specification of the conditional VAE.

Module	Layer	Output dim.	Conditioning
Condition embed	Linear–SiLU–Linear	64	—
Null embedding	Learned parameter	64	replaces embed when dropped
Encoder input	Linear	256	—
Encoder blocks × 3	Linear–SiLU–Dropout + skip	256	FiLM(**h**, **c**)
Latent heads	Linear (***μ***), Linear (log***σ***^2^)	32	—
Decoder input	Linear	256	—
Decoder blocks × 3	Linear–SiLU–Dropout + skip	256	FiLM(**h**, **c**)
Decoder output	Linear	32	—

FiLM parameters ***γ***, ***β*** are produced by independent linear maps ℝ^64^ → ℝ^256^ per block.

### 3.6 Inverse-design validation protocol

Inverse design was evaluated against four operating-point specifications derived from the empirical PV-target distribution, as listed in Table 2.

**Table 2:** Target operating-point specifications used for inverse-design evaluation.

Operating point	*V*_oc_ (V)	*J*_sc_ (A m^−2^)	*FF*	PCE_proxy_
Median device	1.265	−212.31	0.740	0.192
High-PCE (90th pct)	1.351	−216.14	0.876	0.246
Top-tier (99th pct)	1.374	−217.93	0.903	0.261
Ultra (101% of top)	1.388	−220.11	0.912	0.264

For each operating point, 1, 000 candidate devices were generated using the cVAE with the optimal CFG scale, and three baseline conditions were compared: (i) cVAE+CFG (proposed), (ii) cVAE without guidance (*w* = 0), and (iii) random sampling of real device parameters from the training set. The hit-rate is defined as Equation 17


$$\begin{array}{l} HR\left(c^{*}\right)=\frac{1}{N_{\text{gen}}}\underset{j}{\sum }1\left[\bar{\text{PCE}}\left(x_{j}^{\text{gen}}\right)\geq c_{\text{PCE}}^{*}\right]. \end{array}$$ (17)

Physical plausibility of generated devices was further verified via two-sample Kolmogorov–Smirnov tests between the marginal distributions of every feature in the real and generated populations.

## 4 Results and discussion

### 4.1 Exploratory analysis of the dataset

**Figure 3:**
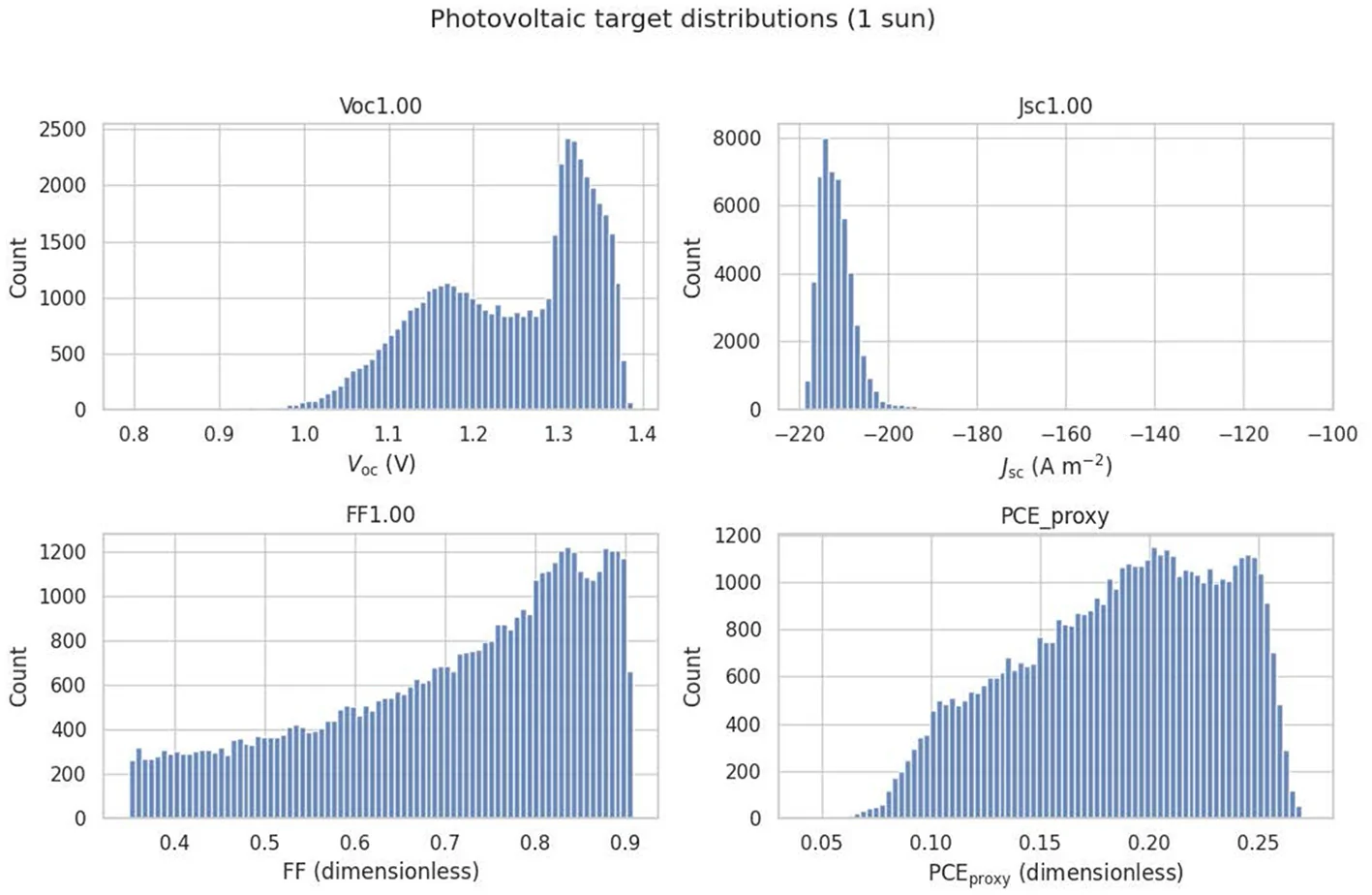
Distributions of the photovoltaic targets (*V*_oc_, *J*_sc_, *FF*, PCE_proxy_) over the 49, 998-device dataset.

**Figure 4:**
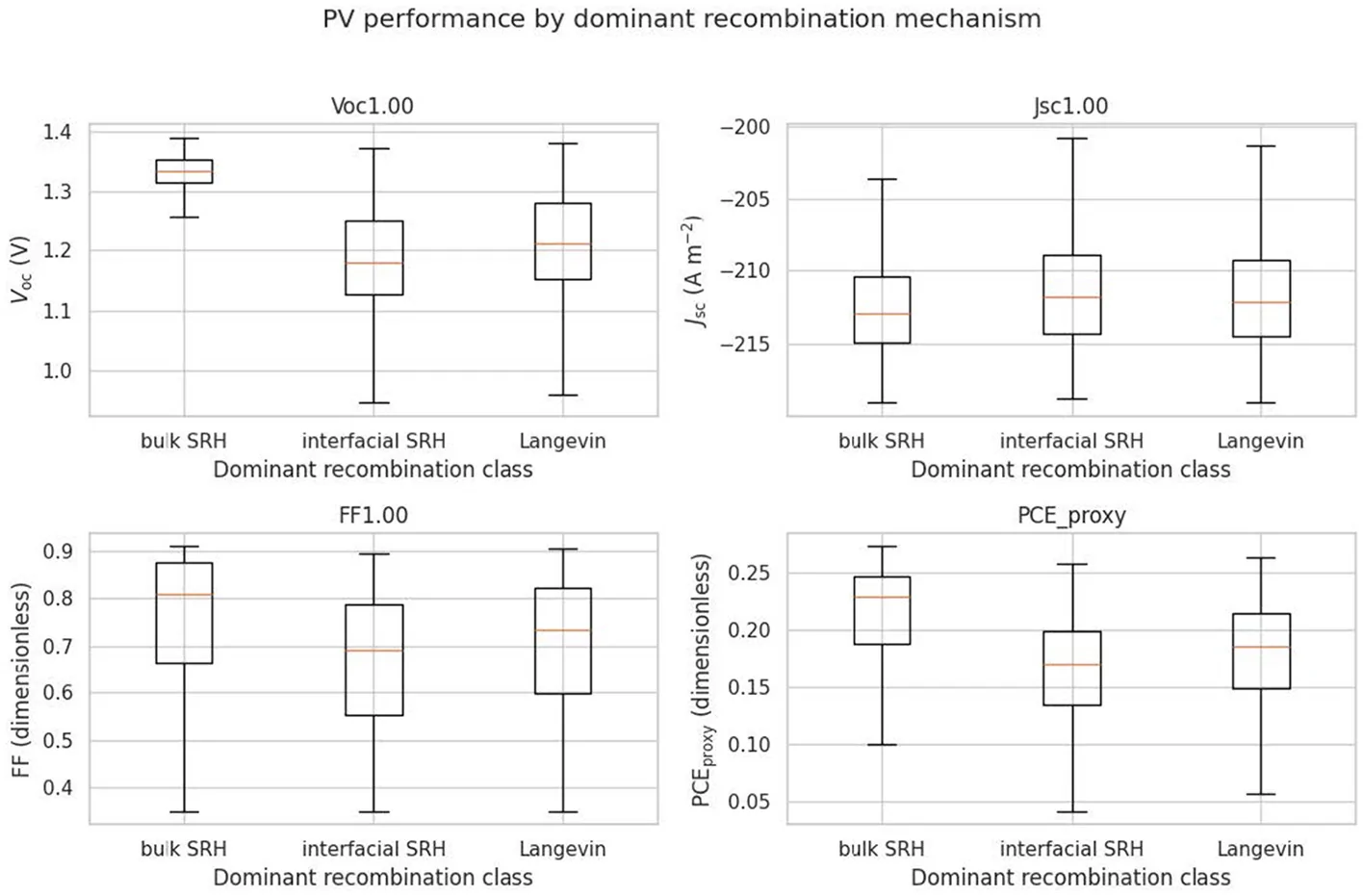
Boxplots of *V*_oc_, *FF* and PCE_proxy_ stratified by dominant recombination class (0: bulk SRH, 1: interfacial SRH, 2: Langevin bimolecular).

**Figure 5:**
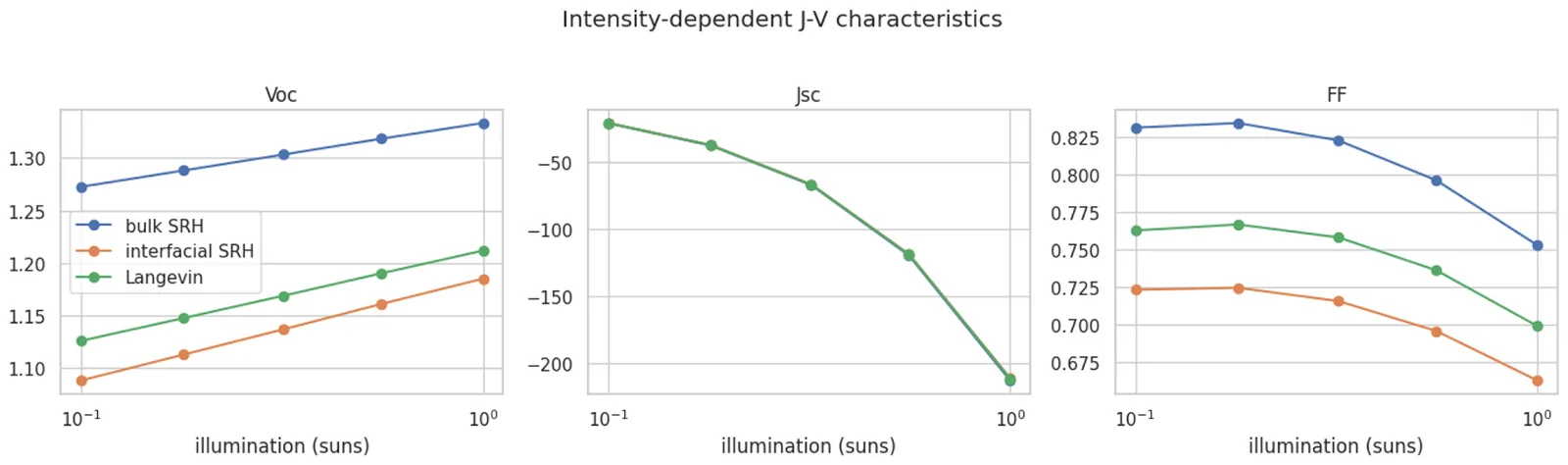
Mean (± std) of *V*_oc_, *J*_sc_, and *FF* as functions of light intensity from 0.10 to 1.00 suns. The expected logarithmic dependence of *V*_oc_ and approximately linear dependence of *J*_sc_ are reproduced.

**Figure 6:**
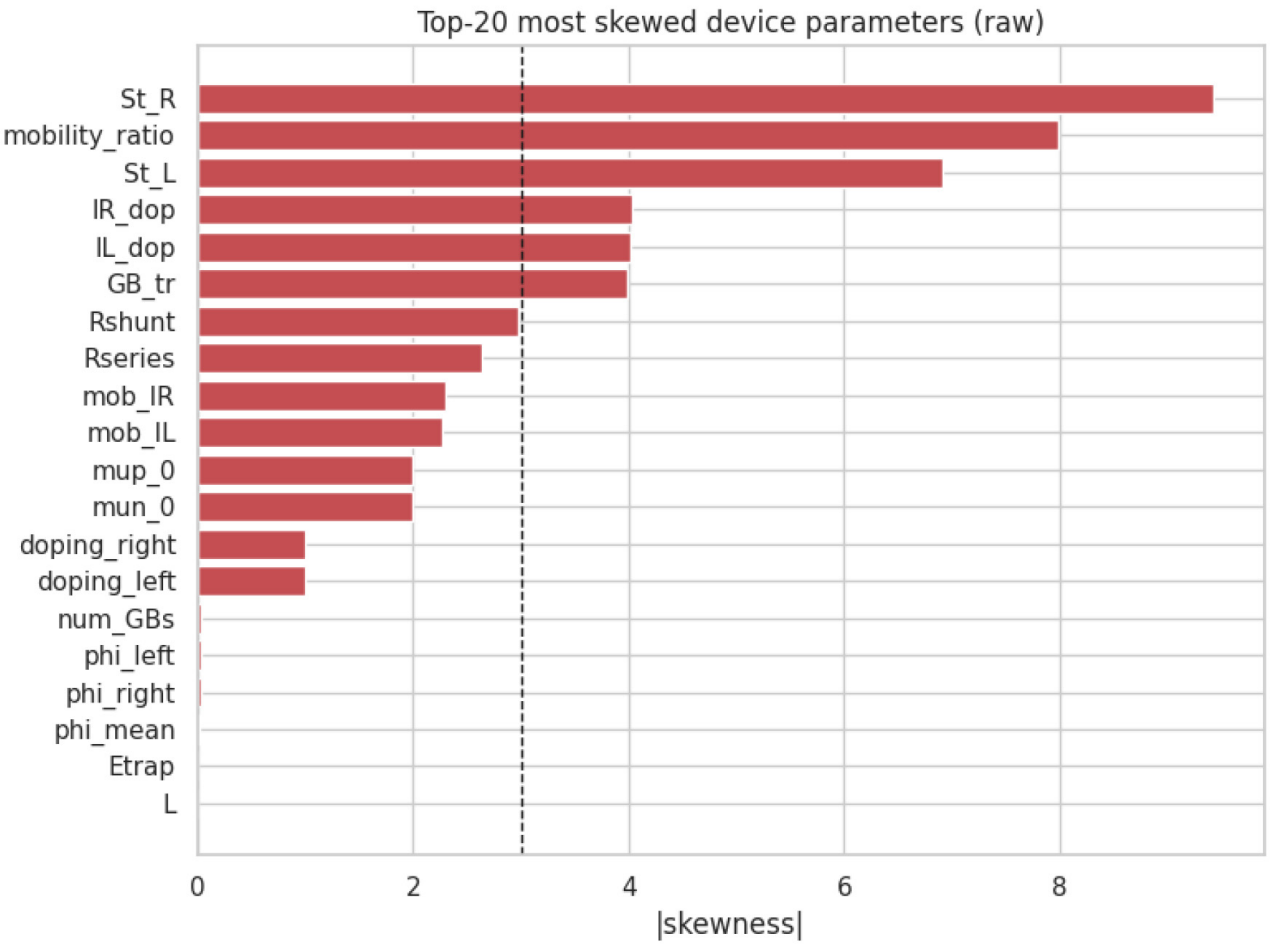
Top-20 most skewed device parameters, motivating the signed-logarithmic transform.

**Figure 7:**
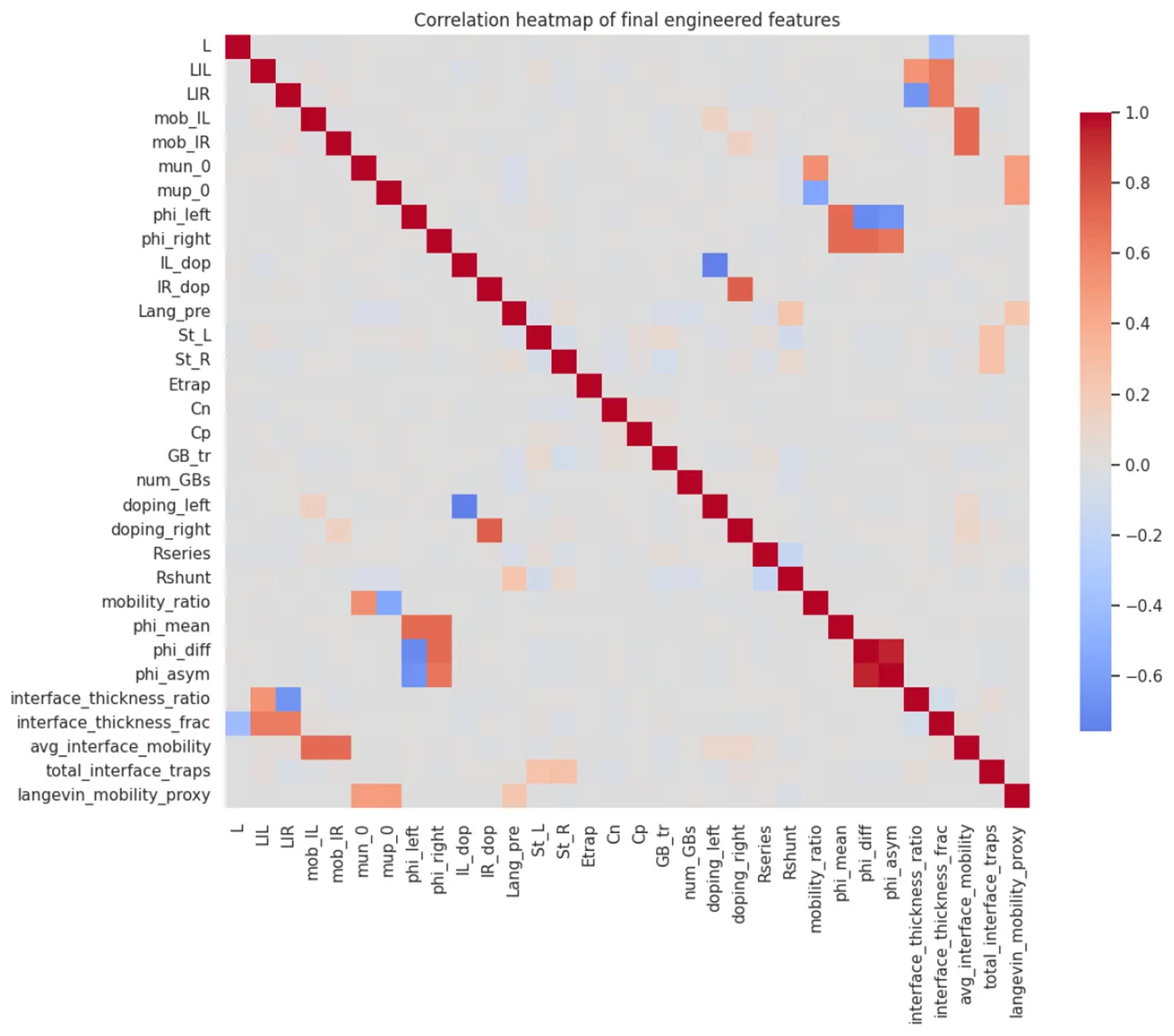
Correlation heatmap of the final 32-dimensional engineered feature vector after Pearson redundancy filtering.

The dataset comprised 49, 998 records with no missing values or duplicates and an exactly balanced label distribution across the three recombination-mechanism classes (16, 666 samples each). Figures 3–5 present, respectively, the marginal distributions of the four photovoltaic targets, their dependence on the dominant recombination class, and the systematic shift of *V*_oc_, *J*_sc_, *FF* across five illumination intensities. The skewness analysis (Figure 6) confirmed the need for the signed-logarithmic transform of Equation 2, while the post-engineering correlation map (Figure 7) demonstrates that the engineered descriptors carry distinct information after redundancy filtering.

### 4.2 Mutual-information feature ranking

**Figure 8:**
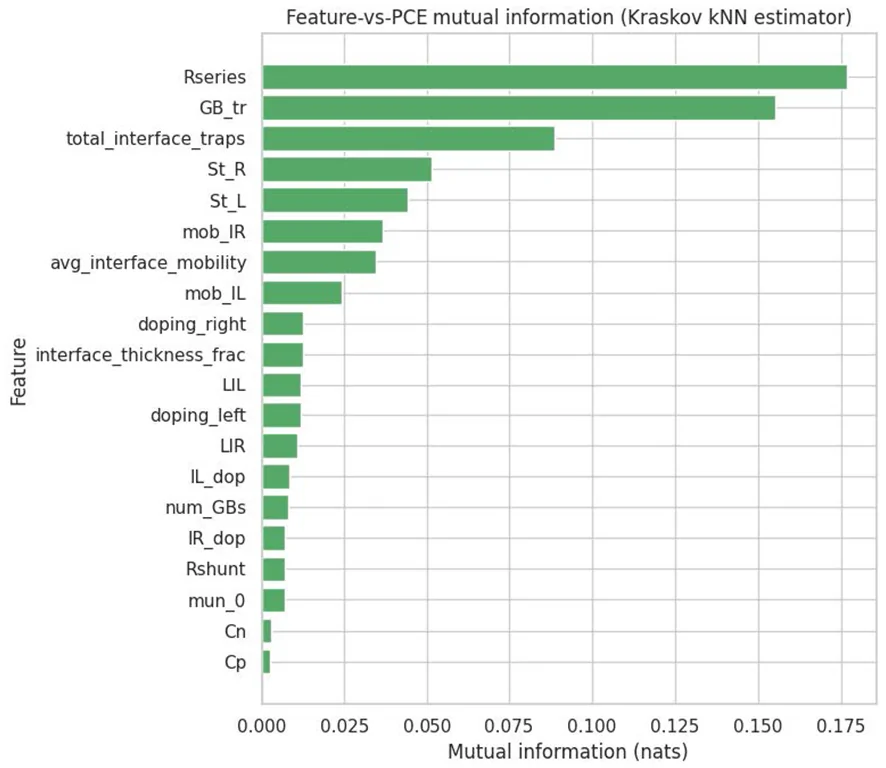
Mutual information between every input feature and PCE_proxy_, estimated using the Kraskov *k*-nearest-neighbor estimator.

The Kraskov mutual-information estimator (Equation 11) was applied to each feature against the PCE proxy. The top-ranked features (Figure 8) are listed with physical interpretation in Table 3.

**Table 3:** Top-10 device descriptors ranked by mutual information with the PCE_proxy_ target.

Rank	Feature	Mutual information	Physical interpretation
1	Rseries	0.1769	Parasitic series resistance—*FF*/*V*_oc_ loss
2	GB_tr	0.1553	Grain-boundary defect density—bulk recomb.
3	total_interface_ traps	0.0885	Sum of left+right interface trap densities
4	St_R	0.0517	Right-interface trap density
5	St_L	0.0442	Left-interface trap density
6	mob_IR	0.0365	Right interface mobility
7	avg_interface_ mobility	0.0343	Mean interface mobility
8	mob_IL	0.0242	Left interface mobility
9	doping_right	0.0125	Right transport-layer doping
10	interface_ thickness_frac	0.0125	Interface-to-absorber thickness fraction

### 4.3 Forward surrogate performance

Table 4 reports the five-fold cross-validation performance of the seven forward surrogate models. Gradient-boosted tree ensembles dominate the ranking, consistent with the tabular-learning literature (Grinsztajn et al., 2022; McElfresh et al., 2023): XGBoost attained the highest mean *R*^2^ (0.8661 ± 0.0020), statistically tied with LightGBM (0.8661 ± 0.0021), followed by gradient boosting (0.8516) and the randomized-tree ensembles. The multilayer perceptron trailed the tree ensembles (*R*^2^ = 0.7178 ± 0.0134), and Ridge regression—a linear baseline—lagged substantially (*R*^2^ = 0.4607), confirming the strongly non-linear nature of the structure–property mapping.

**Table 4:** Five-fold cross-validation results of the forward surrogate models on the PCE_proxy_ target.

Model	*R*^2^ (mean ±std)	RMSE (mean)	MAE (mean)	Rank
**XGBoost**	**0.8661 ± 0.0020**	**0.0172**	**0.0127**	1
LightGBM	0.8661 ± 0.0021	0.0172	0.0127	2
GradBoost	0.8516 ± 0.0023	0.0181	0.0135	3
RandomForest	0.8301 ± 0.0022	0.0193	0.0144	4
ExtraTrees	0.8237 ± 0.0012	0.0197	0.0147	5
MLP	0.7178 ± 0.0134	0.0249	0.0189	6
Ridge	0.4607 ± 0.0039	0.0344	0.0276	7

Ranking is by mean out-of-fold *R*^2^. Bold values indicate the best-performing model on each metric.

**Figure 9:**
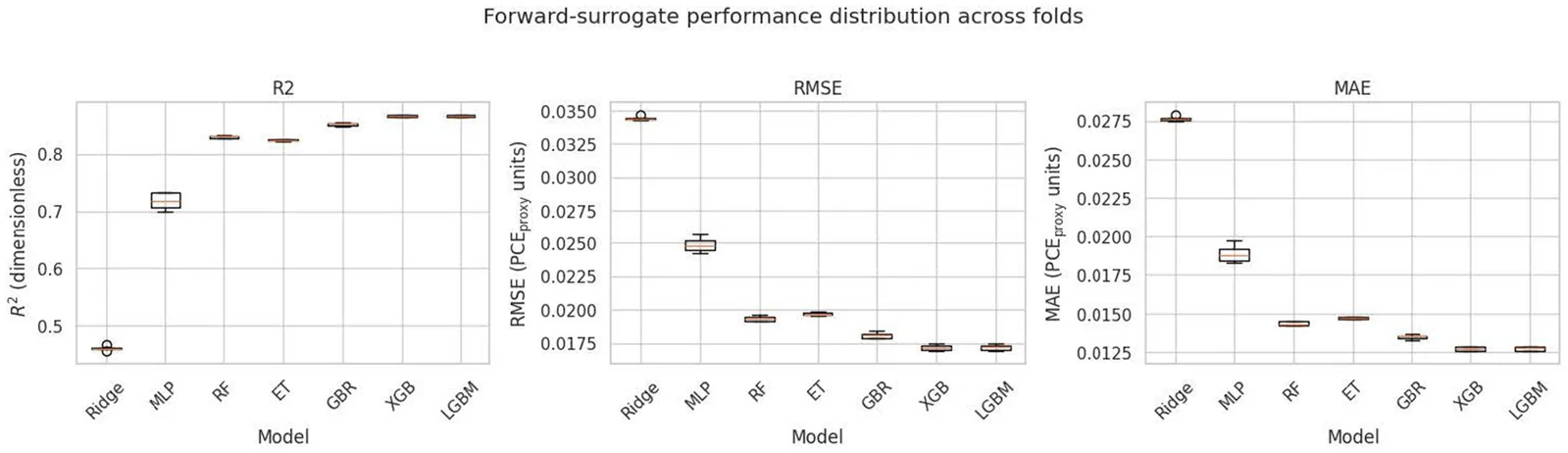
Distributions of *R*^2^, RMSE, and MAE across five cross-validation folds for the seven forward-surrogate models.

**Figure 10:**
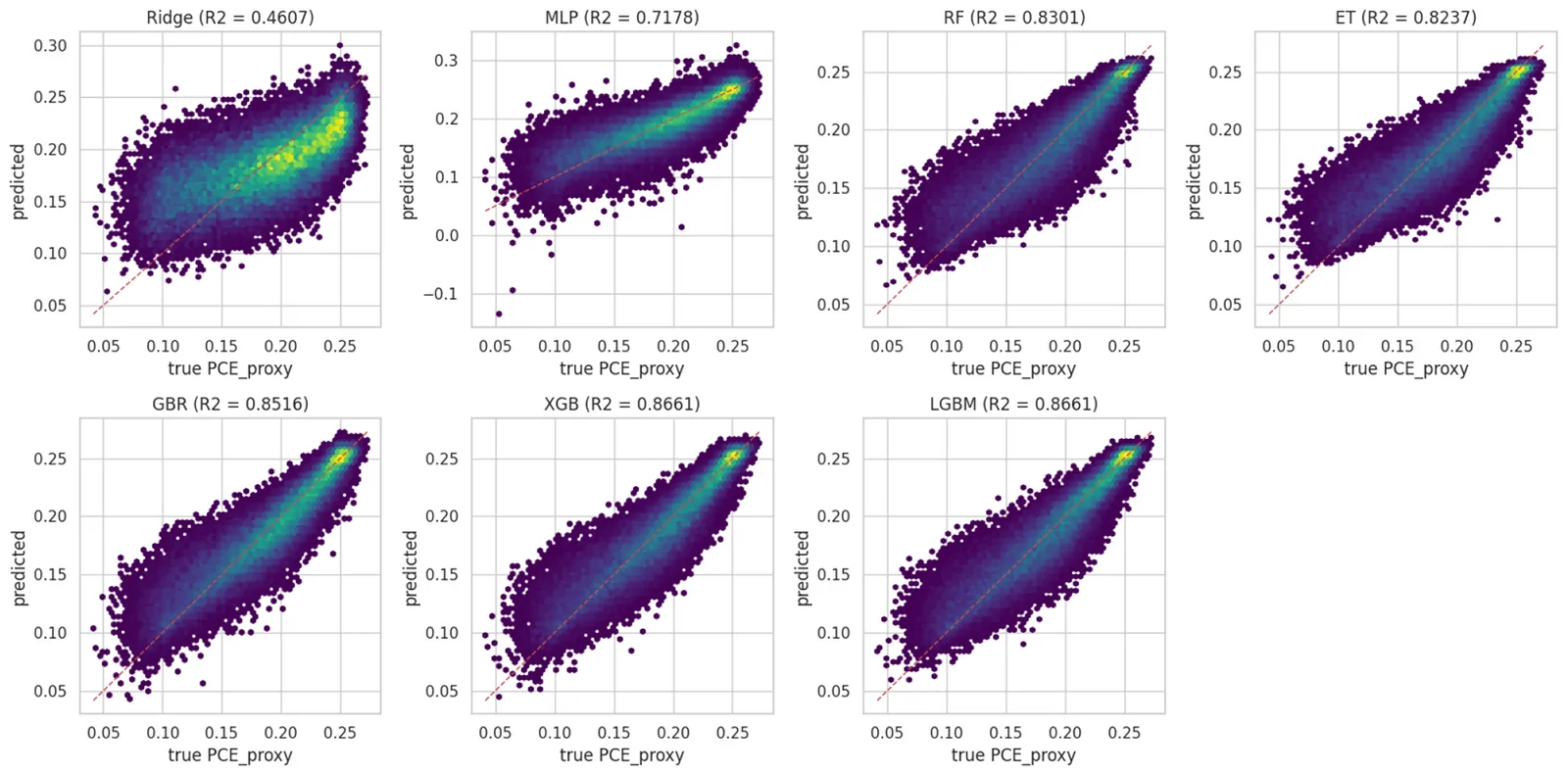
Out-of-fold parity plots (predicted vs. true PCE_proxy_) for each forward-surrogate model.

**Figure 11:**
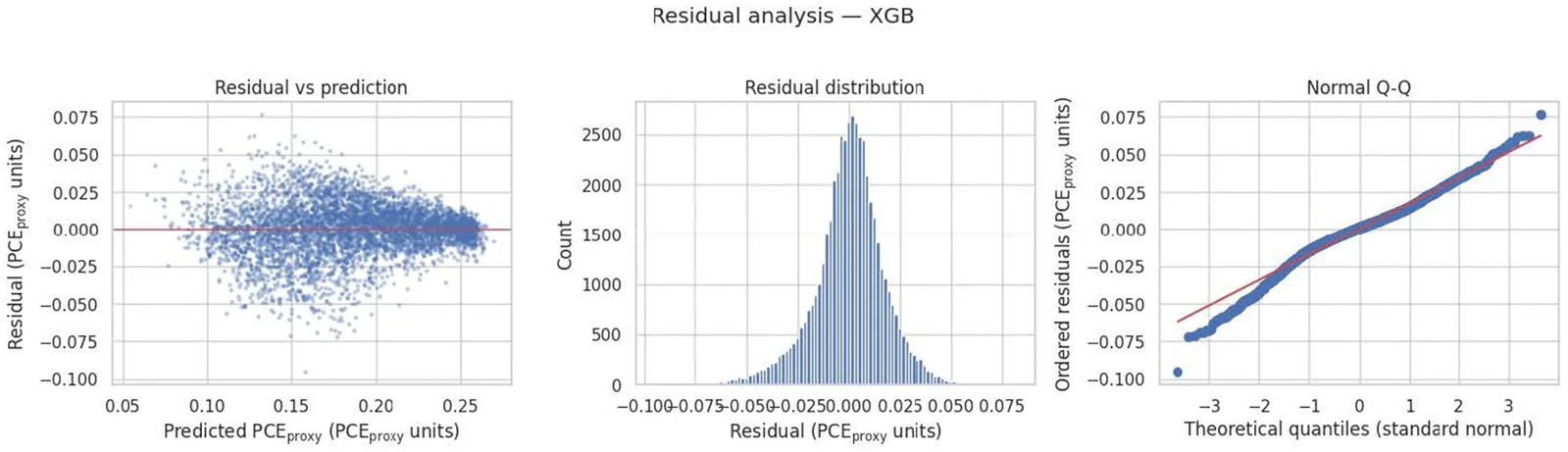
Residual analysis for the best forward surrogate (XGBoost): residuals vs. predicted values, residual distribution, and Q–Q plot.

The tight fold-to-fold standard deviations (below 0.3% of the mean *R*^2^ for the tree ensembles) reflect the stability of the leakage-controlled protocol, in which the quantile normalizer is refitted on each training fold and applied unseen to the held-out fold. The XGBoost, LightGBM, and gradient-boosting models were selected to constitute the three-member oracle ensemble (Equation 8), whose members reach held-out *R*^2^ of 0.863, 0.864, and 0.847 on the fixed 80/20 validation split used for inverse design. Boxplots of per-fold metrics (Figure 9), parity plots of out-of-fold predictions (Figure 10), and residual diagnostics for the best model (Figure 11) corroborate these conclusions.

### 4.4 Statistical comparison of surrogates

**Figure 12:**
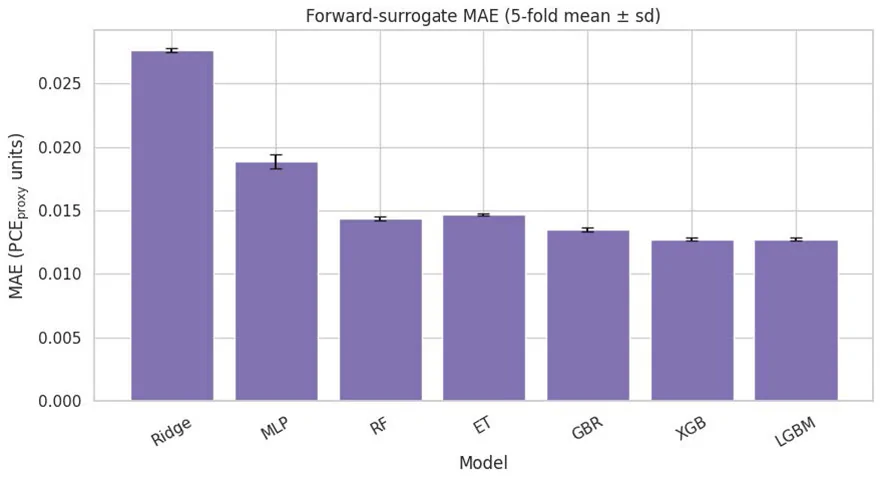
Mean absolute error per surrogate model (5-fold mean ± sd). Statistical significance and effect sizes of pairwise differences are reported in Table 5.

Table 5 reports pairwise Wilcoxon signed-rank and paired-*t* tests on the out-of-fold absolute-error vectors between XGBoost and each competitor, together with effect-size measures that quantify *practical* significance alongside statistical significance. Five of the six comparisons are statistically significant at α = 0.05 with *p*-values many orders of magnitude below threshold. The single exception is instructive: XGBoost and LightGBM are statistically indistinguishable (Wilcoxon *p* = 0.51, paired-*t* *p* = 0.92, rank-biserial *r* = 0.003), which is expected for two well-tuned gradient-boosting implementations of the same inductive bias and motivates retaining both in the oracle ensemble rather than declaring an artificial winner. Figure 12 summarizes mean absolute error by model.

**Table 5:** Pairwise statistical comparison of the XGBoost surrogate against each competitor on out-of-fold absolute errors, with effect sizes (Cohen's *d* on paired differences; rank-biserial correlation *r*).

Comparison (XGB vs.)	${\bar{\left|\text{err}\right|}}_{\text{XGB}}$	${\bar{\left|\text{err}\right|}}_{\text{other}}$	Wilcoxon *p*	Paired-*t* *p*	*d*	*r*	Sig.
Ridge	0.0127	0.0276	< 10^−300^	< 10^−300^	0.72	0.70	✓
MLP	0.0127	0.0189	< 10^−300^	< 10^−300^	0.42	0.45	✓
RandomForest	0.0127	0.0144	< 10^−300^	< 10^−300^	0.20	0.21	✓
ExtraTrees	0.0127	0.0147	< 10^−300^	< 10^−300^	0.23	0.24	✓
GradBoost	0.0127	0.0135	8.01 × 10^−193^	1.49 × 10^−185^	0.13	0.15	✓
LightGBM	0.0127	0.0127	0.51	0.92	0.00	0.00	n.s.

Bold checkmarks (✓) indicate statistically significant differences at α = 0.05; ‘n.s.' denotes not significant. |err| values are mean absolute errors on out-of-fold predictions. Cohen's d effect sizes: small (0.2), medium (0.5), large (0.8).

Because *N*≈5 × 10^4^ paired samples render even minute differences statistically detectable, effect sizes carry the practical interpretation: the XGBoost advantage over the strongest non-tied competitor (GradBoost) amounts to ΔMAE = 7 × 10^−4^ PCE units (0.39% of the median PCE proxy; Cohen's *d* = 0.13, small), whereas the advantages over the MLP and Ridge baselines are practically meaningful (3.2% and 7.8% of the median PCE; *d* = 0.42 and 0.72).

Because the Shapiro–Wilk normality test on error differences rejected normality (*p* < 10^−22^) in every comparison, the non-parametric Wilcoxon result dominates interpretation; the parametric *t*-test corroborates every conclusion.

### 4.5 Explainable AI: drivers of PCE

**Figure 13:**
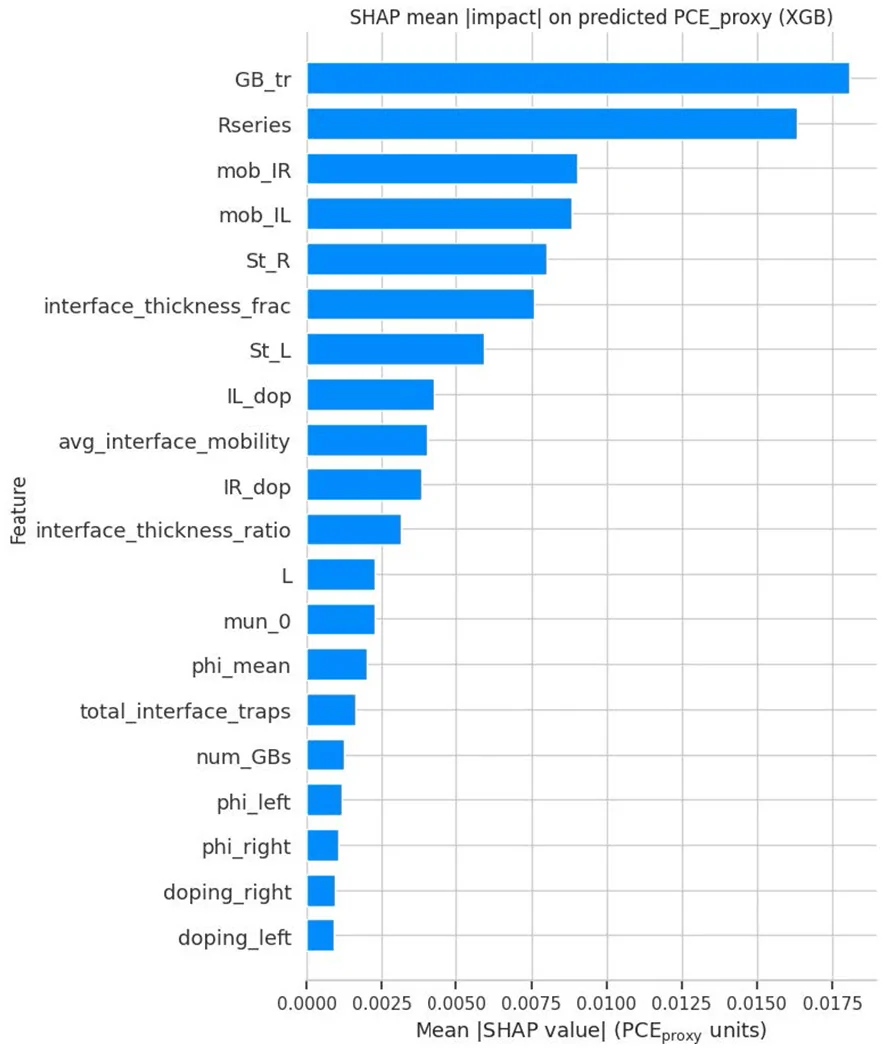
SHAP summary plot showing the mean absolute Shapley value (i.e., mean impact on the predicted PCE_proxy_) for each input feature.

**Figure 14:**
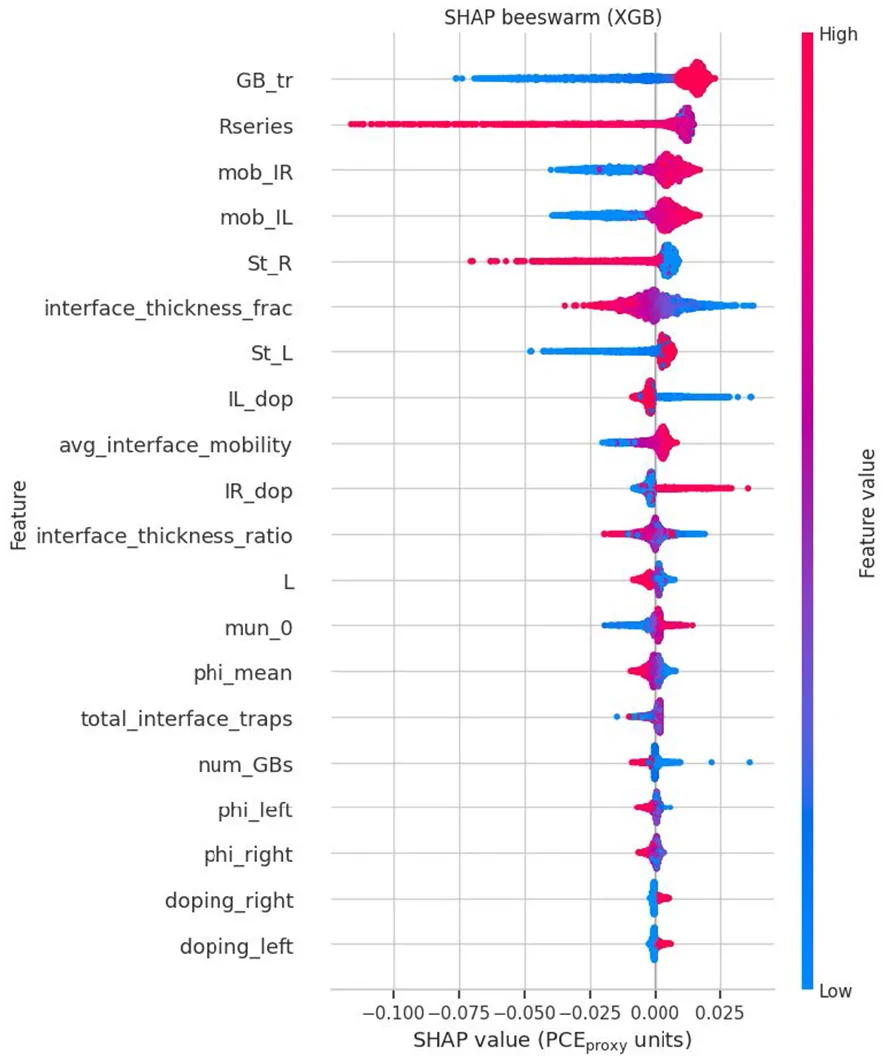
SHAP beeswarm plot. Each dot is one sample; color encodes the value of the feature, and the horizontal axis encodes its Shapley contribution to the predicted PCE_proxy_.

**Figure 15:**
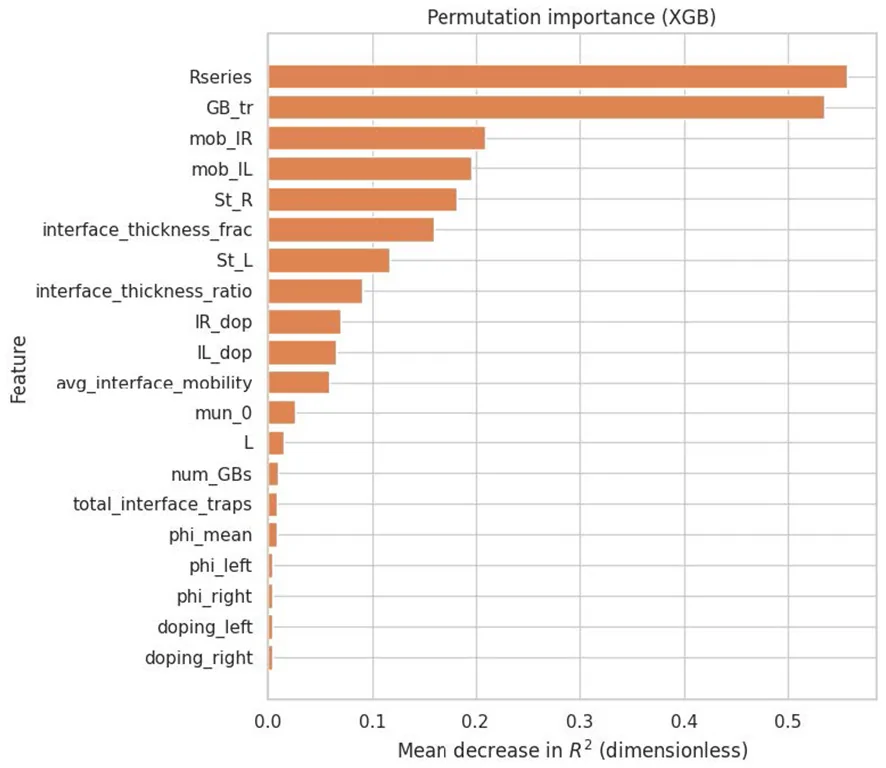
Top-20 permutation importances (decrease in *R*^2^ when each feature is randomly permuted). Error bars indicate the standard deviation across five permutation repeats.

Figure 13 (SHAP mean-|ϕ| summary), Figure 14 (SHAP beeswarm), and Figure 15 (permutation importance) collectively identify the dominant device-physics drivers of the PCE proxy. The three attribution methods converge on the same leading pair: parasitic series resistance (*R*_series_: mutual information *I* = 0.177, permutation importance 0.557, SHAP mean-|ϕ| = 0.0163) and grain-boundary defect density (GB_tr: *I* = 0.155, permutation 0.535, SHAP 0.0181), followed by the interface mobilities (mob_IR, mob_IL), the interfacial trap densities (St_R, St_L), and the interface-to-absorber thickness fraction. These findings reproduce well-established device-physics intuition for polycrystalline halide perovskites (Stranks, 2017; Liu et al., 2023) and provide direct, actionable guidance for experimental prioritization. A class-resolved attribution analysis, which tests whether these global rankings persist within each recombination-mechanism regime, is presented in Section 4.10.

### 4.6 cVAE training dynamics

**Figure 16:**
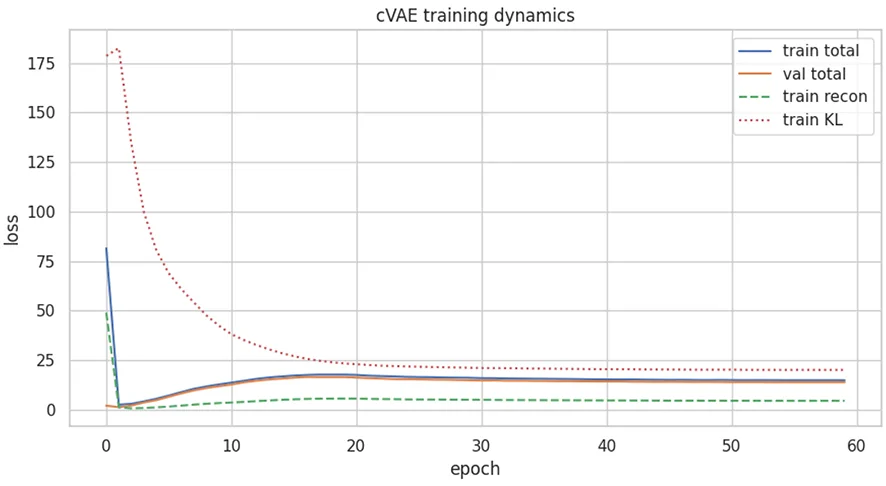
CVAE training and validation loss curves over 60 epochs, with KL annealing controlled by a cosine schedule for β.

The cVAE training trajectory (Figure 16) exhibited the expected three-phase pattern: an early reconstruction-dominated phase (epochs 1–10) with β rising from 0 toward ~0.2 and reconstruction loss falling sharply; a mid-training regularization phase (epochs 10–20) during which β saturates at its terminal value of 0.5 and the total loss transiently rises as the KL term is fully engaged; and a converged plateau (epochs 20–60) where train and validation losses stabilized at 14.81 and 13.81, respectively. The validation loss tracks the training loss closely throughout (the validation objective is evaluated without condition dropout, which is why it sits marginally below the training curve), indicating no overfitting.

### 4.7 Classifier-free guidance: effect of guidance scale

**Figure 17:**
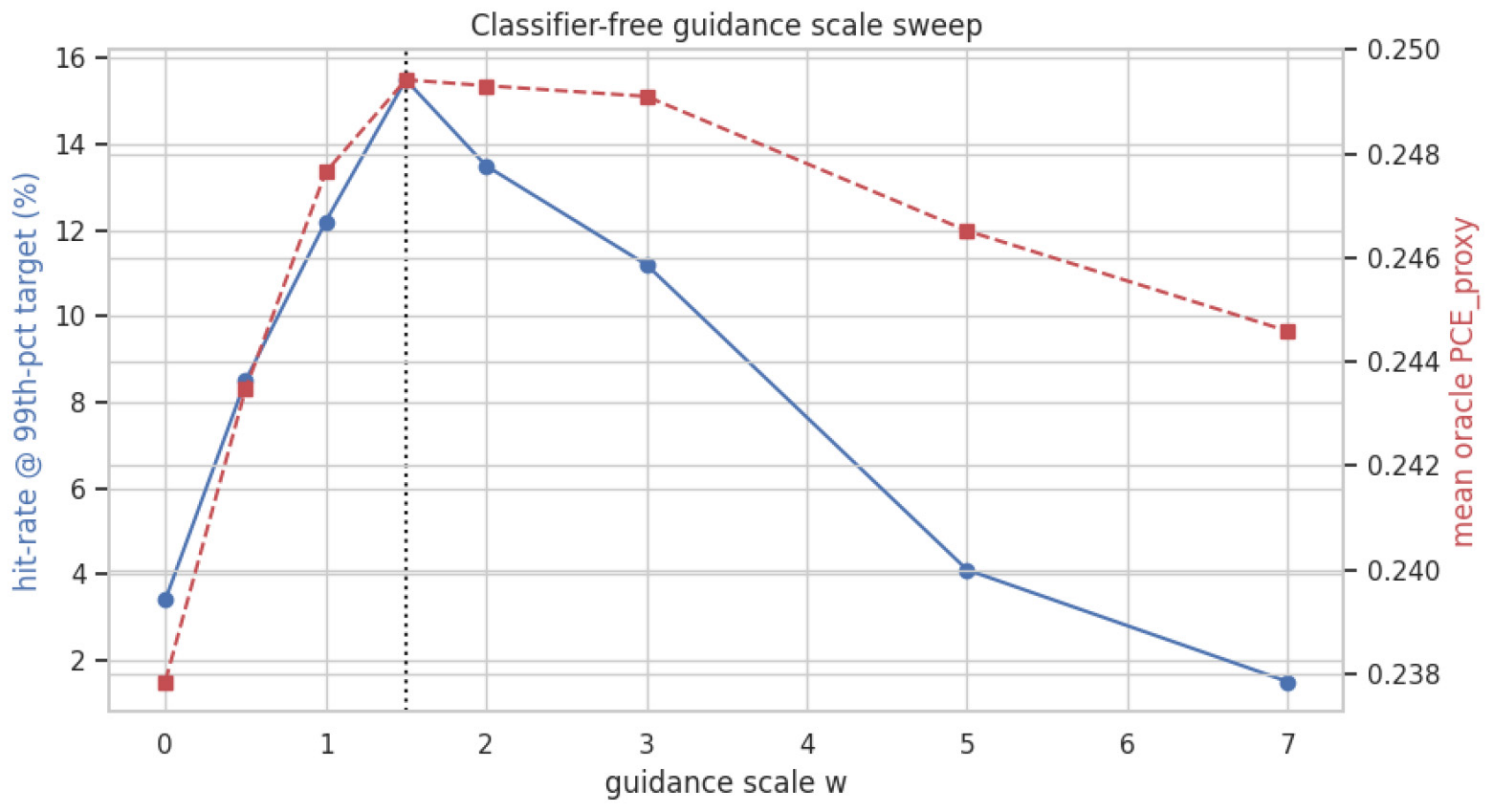
Classifier-free-guidance scale sweep at the Top-tier target: hit-rate **(left axis)** and mean oracle-predicted PCE **(right axis)** as functions of the guidance scale *w*; the dotted vertical line marks the optimum guidance scale *w* = 1.5, where the asterisk (^*^) denotes the value that maximized the hit-rate at the 99th-percentile target.

Table 6 and Figure 17 present the CFG guidance-scale sweep for the Top-tier (99th percentile) target. The hit-rate rises steeply from 3.4% at *w* = 0 (ordinary conditional sampling) to a maximum of 15.5% at *w* = 1.5, then declines as *w* increases further: beyond the optimum, the linear extrapolation of Equation 14 pushes candidates off the physically plausible manifold faster than it gains target fidelity, and the oracle correspondingly scores them lower. This non-monotonic pattern is the classical CFG trade-off between target fidelity and sample realism. The stability of the optimum w^*^ = 1.5 across random seeds, data splits and target operating points is quantified in Section 4.11.

**Table 6:** Classifier-free-guidance scale sweep at the Top-tier (99th percentile) operating point (1, 000 candidates per setting).

CFG scale *w*	$\bar{\text{PCE}}$	Hit-rate
0.0	0.2379	3.4%
0.5	0.2435	8.5%
1.0	0.2476	12.2%
**1.5**	**0.2494**	**15.5%**
2.0	0.2493	13.5%
3.0	0.2491	11.2%
5.0	0.2465	4.1%
7.0	0.2446	1.5%

Bold row indicates the optimal guidance scale (*w* = 1.5) with the highest hit-rate.

### 4.8 Inverse-design performance against baselines

Table 7 summarizes the central inverse-design result. Across all four operating-point specifications, the proposed cVAE+CFG framework outperforms both the unguided cVAE and random sampling, and the *relative* advantage grows monotonically as the target moves into the distribution tail: from 1.3 × over random

**Table 7:** Inverse-design hit-rate (HR) of the proposed cVAE+CFG (w^*^ = 1.5) against unguided cVAE (*w* = 0) and random sampling of real training devices (1, 000 candidates per condition).

Operating point	Target PCE	HR_CFG_	HR_uncond_	HR_random_	Lift
Median device	0.1919	61.4%	55.9%	48.1%	1.3 ×
High-PCE (90th pct)	0.2460	73.7%	39.0%	8.3%	8.9 ×
**Top-tier (99th pct)**	**0.2609**	**12.6%**	3.0%	0.5%	**25.2 × **
**Ultra (101%** **of top)**	**0.2635**	**3.4%**	1.4%	0.0%	**≥34 × **

**Figure 18:**
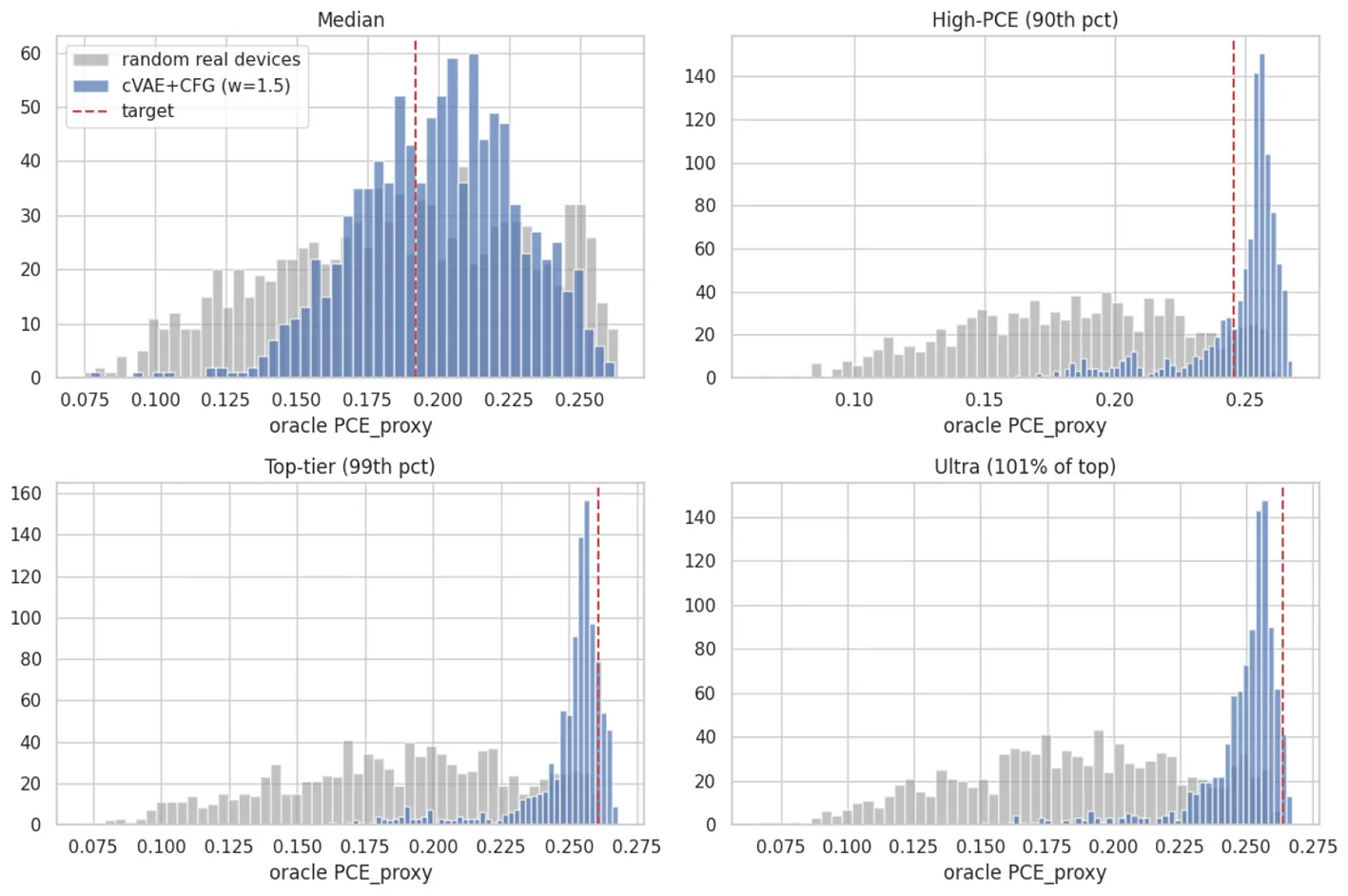
Histograms of oracle-predicted PCE for cVAE+CFG (proposed) vs. random sampling, across the four target operating points. The dashed red line indicates the target PCE.

The rightmost column reports the lift over random; at the Ultra target the lift is a lower bound because random sampling scored 0/1, 000. Bold rows indicate the tail-region operating points (Top-tier and Ultra) where the proposed method provides the largest relative gains.

at the median target, to 8.9 × at the 90th percentile, 25.2 × at the 99th percentile, and at least 34 × at the Ultra target, where random sampling of real training devices produced zero hits in 1, 000 draws (the lift is therefore a lower bound computed against the resolution floor of 1/1, 000). Absolute hit-rates decrease toward the tail for all methods, as expected for targets of vanishing empirical support; the value of guidance lies precisely in multiplying access to this tail. Figure 18 visualizes the corresponding distributions of oracle-predicted PCE: guided generation shifts the entire distribution toward the target rather than merely stretching its upper tail.

Two aspects of Table 7 deserve emphasis. First, the unguided cVAE already concentrates samples toward the conditioning vector (it is a *conditional* model), yet guidance multiplies its tail hit-rate by a factor of 2–4 at the two highest targets, demonstrating that CFG is the operative mechanism for tail-region generation rather than a simple conditioning bias. Second, all reported hit-rates are computed with the learned oracle ensemble (Equation 8); at the Ultra target in particular, the oracle itself operates in an extrapolative regime where its predictive error is unquantified. The reported tail hit-rates should therefore be read as oracle-certified rates pending drift-diffusion re-simulation, which we address explicitly in Section 4.14 by exporting the top-ranked candidates in physical units for direct re-validation in the simulator.

### 4.9 Physical plausibility of generated devices

**Figure 19:**
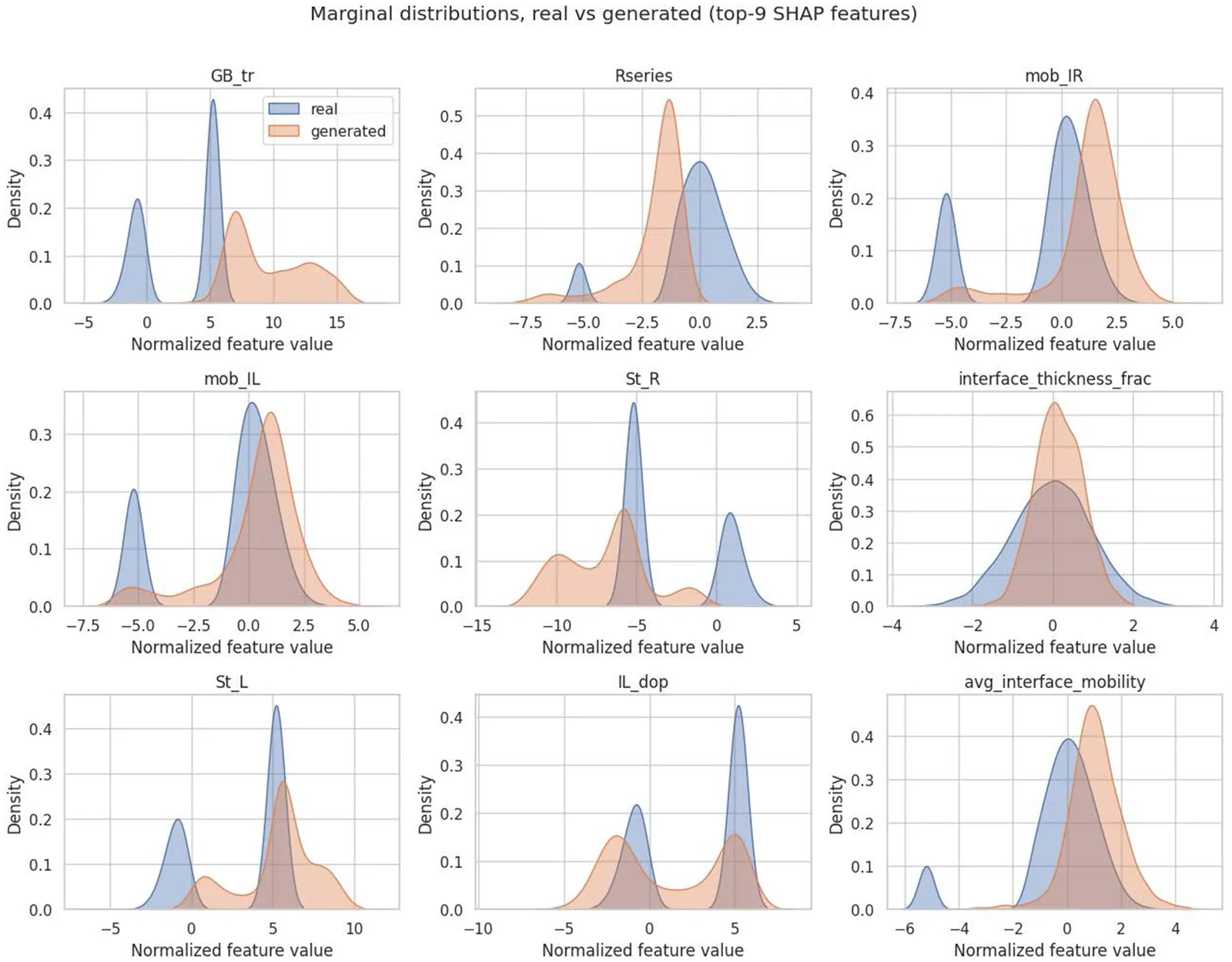
Kernel-density estimates of the marginal distributions of the top-9 important features in the real (blue) and cVAE+CFG-generated (red) populations, conditioned on the Top-tier target.

Two-sample Kolmogorov–Smirnov tests on the marginals of real vs. generated device parameters at the Top-tier target (Table 8 and Figure 19) show a physically coherent picture. The energy-level descriptors are the most faithfully preserved marginals: the work-function asymmetry is statistically indistinguishable from the real distribution (KS = 0.029, *p* = 0.48), followed by the bulk trap energy Etrap (KS = 0.054) and the work-function difference and left work function (KS ≤ 0.14). Conversely, the parameters that the explainability analysis identified as performance-limiting—trap densities, dopings, mobilities and parasitic resistances—exhibit strongly shifted marginals (KS up to ≈0.4), and overall only 1 of 32 features retains *p*>0.05. This is the expected and indeed desired behavior of a *guided* generator: when conditioned on a 99th-percentile target, the model must abandon a representative cross-section of the joint distribution and concentrate probability mass on the high-performance sub-manifold, shifting precisely those marginals that control performance while respecting the energy-level structure of the device.

**Table 8:** Kolmogorov–Smirnov tests on marginal distributions of representative features (real vs. generated, Top-tier target).

Feature	KS statistic	*p*-value	Class
phi_asym	0.029	0.479	Best-matched
Etrap	0.054	0.015	
phi_diff	0.092	1.5 × 10^−6^	
phi_left	0.136	7.6 × 10^−14^	
mun_0	0.158	1.5 × 10^−18^	
mup_0	0.181	3.3 × 10^−24^	
mob_IL	0.323	2.3 × 10^−77^	Target-shifted
doping_right	0.389	1.2 × 10^−113^	
IR_dop	0.395	3.6 × 10^−117^	

Only phi_asym retains *p*>0.05; the strongly shifted features coincide with the performance-limiting parameters identified by the XAI analysis.

### 4.10 Class-resolved explainability

**Figure 20:**
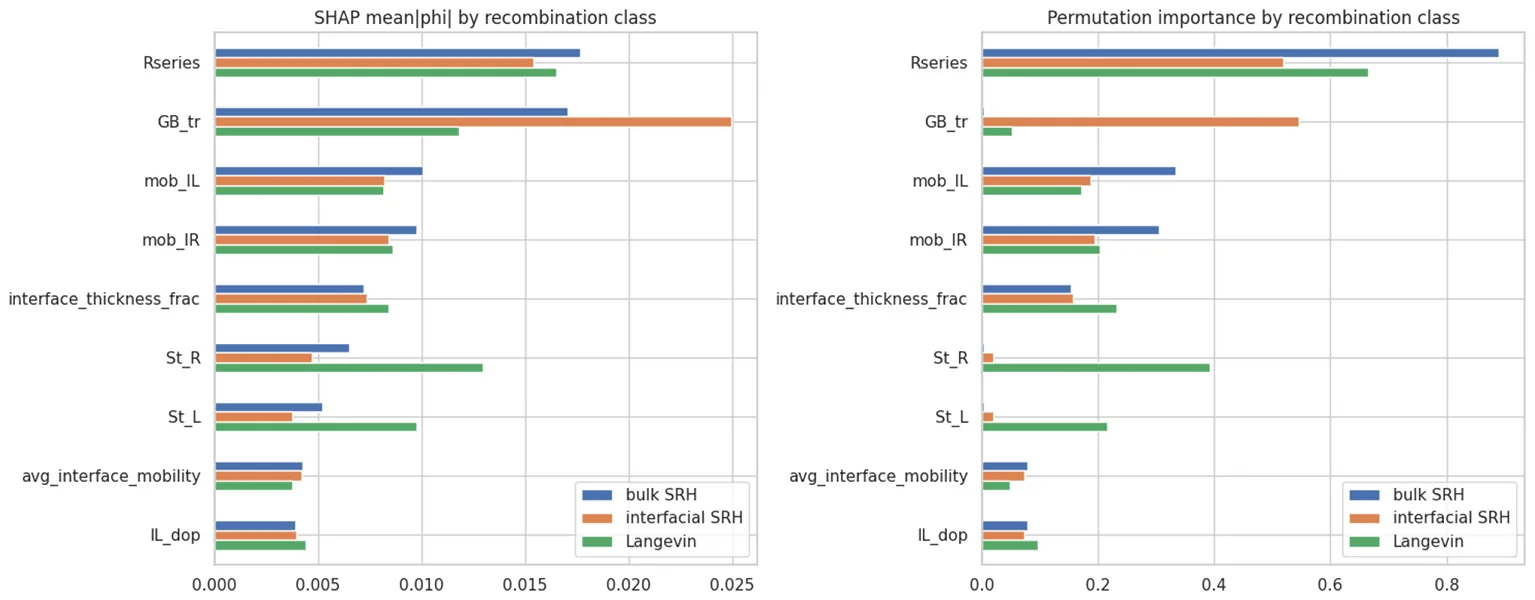
Class-resolved attribution: SHAP mean-|ϕ| (left) and permutation importance (right) computed separately within each recombination-mechanism class for the top-ranked features.

To test whether the global importance rankings persist within each recombination regime, the SHAP and permutation-importance analyses were repeated separately on the bulk-SRH, interfacial-SRH and Langevin sub-populations of the validation set (Figure 20). Series resistance is universal: it ranks first or second by both attribution methods in every class, confirming its status as a mechanism-independent loss channel. The trap-related descriptors, by contrast, are strongly mechanism-specific. Grain-boundary defect density is the top-ranked feature by both methods in the interfacial-SRH class but falls to permutation rank 15 (bulk SRH) and 10 (Langevin) while remaining in the SHAP top-3 in all classes; the right-interface trap density St_R rises to rank 2 by both methods in the Langevin class.

**Figure 21:**
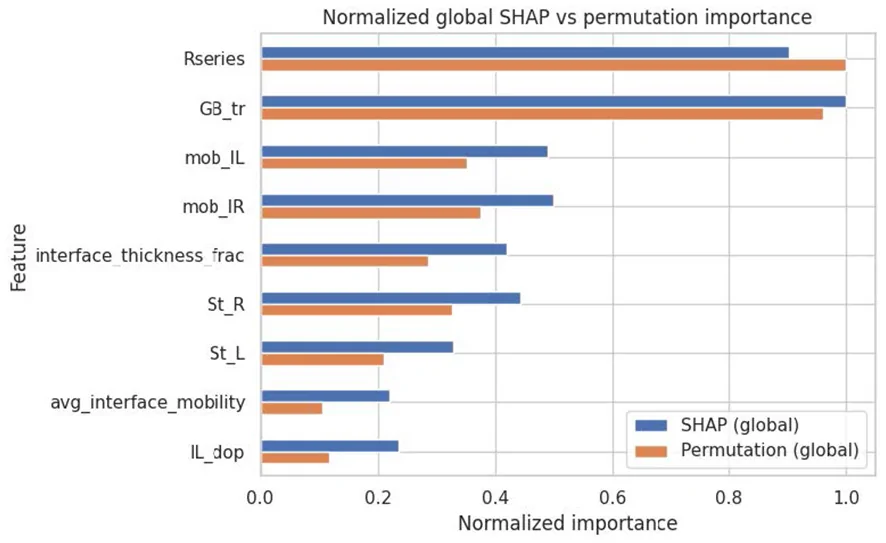
Normalized global SHAP vs. permutation-importance profiles. Agreement on unique-information features (Rseries, mobilities) and divergence on redundancy-affected trap descriptors.

This class dependence also resolves the apparent SHAP-vs.-permutation discrepancy for GB_tr. SHAP distributes credit among features according to their marginal contributions across coalitions, whereas permutation importance measures the performance drop when a single feature is corrupted while all correlated companions remain intact; features whose information is partially recoverable from companions (here, the trap-related descriptors within specific recombination regimes, e.g., the engineered total interface trap density) therefore score systematically lower under permutation than under SHAP (Breiman, 2001; Lundberg and Lee, 2017). The two methods agree closely wherever a feature carries unique information (Rseries, mobilities), and diverge only where redundancy is class-conditional (Figure 21).

### 4.11 Robustness of the guidance-scale optimum

**Figure 22:**
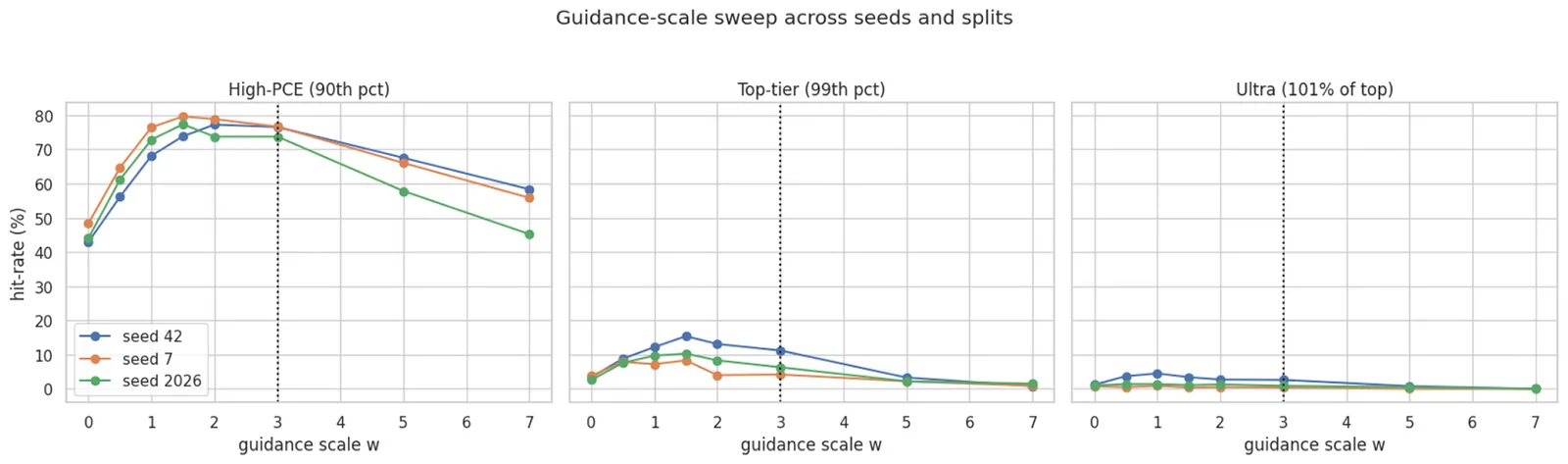
Guidance-scale sweep repeated across three random seeds and independent train/validation splits at three targets. The dotted line marks *w* = 3; the plateau over *w*∈[1, 2] is reproduced by every seed.

Because the guidance scale is the single most consequential inverse-design hyperparameter, the entire cVAE training and guidance sweep was repeated under three independent random seeds (42, 7, 2, 026), each with its own stratified train/validation split, refitted pre-processing, and refitted oracle ensemble (Figure 22). The optimum is highly stable: at the Top-tier target, w^*^ = 1.5 for all three seeds; at the High-PCE target, w^*^∈{1.5, 2.0} (mean 1.67 ± 0.29); and at the extrapolative Ultra target the optimum shifts mildly toward weaker guidance (w^*^∈{0.5, 1.0}), consistent with the general principle that aggressive guidance amplifies off-manifold drift fastest where empirical support is weakest. Across all seeds and targets the hit-rate curves share the same unimodal shape with a broad plateau over *w*∈[1, 2], indicating that the framework does not depend on fine-tuning of *w*.

### 4.12 Architecture sensitivity

**Figure 23:**
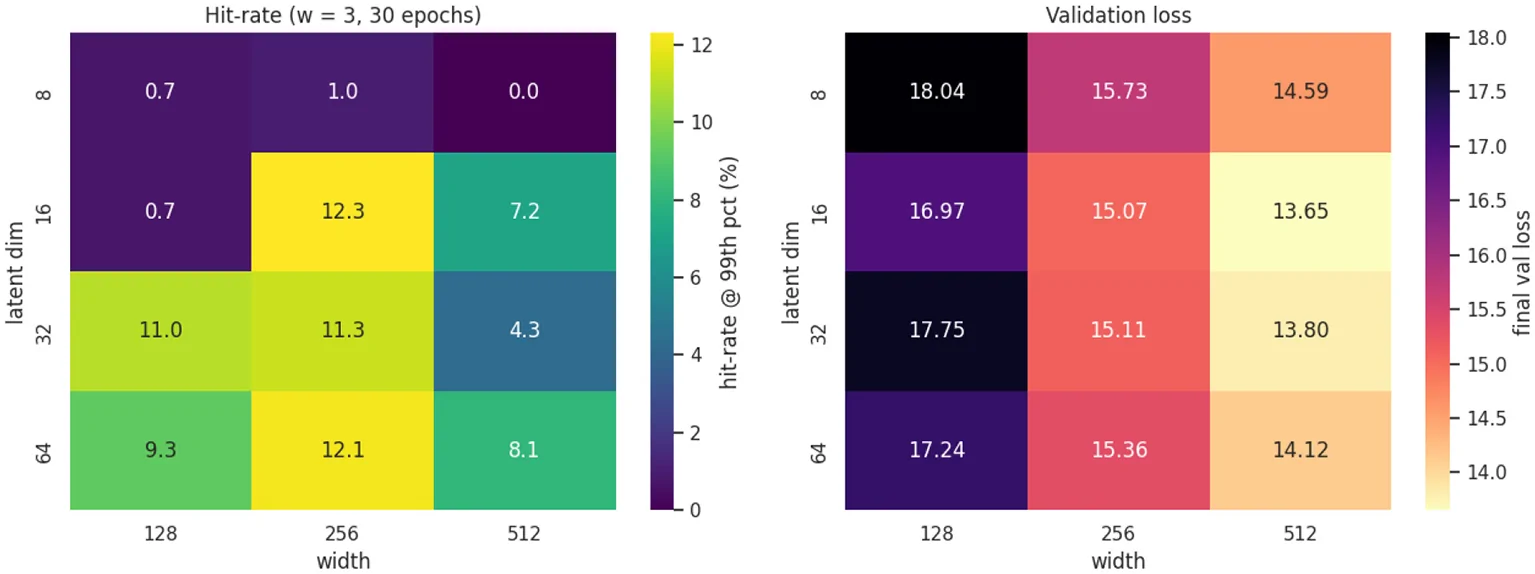
Architecture-sensitivity grid: hit-rate at the Top-tier target **(left)** and final validation loss **(right)** as functions of latent dimension and network width (30-epoch runs).

The latent dimensionality and network width were varied over a 4 × 3 grid (latent ∈{8, 16, 32, 64}, width ∈{128, 256, 512}) with 30-epoch training per cell (Figure 23). Two clear patterns emerge. First, a latent dimension of 8 collapses inverse-design performance (hit-rates ≤ 1%) even though its validation loss remains competitive—reconstruction quality alone is an insufficient criterion for generative usefulness. Second, for latent ≥16 the hit-rate forms a broad plateau (11–12% at the Top-tier target) with the production configuration (latent 32, width 256) lying on it; width 512 lowers validation loss further but degrades hit-rate, indicating mild over-capacity. The production architecture is therefore not a fragile optimum but a representative point on a stable plateau.

### 4.13 Validity, uniqueness and novelty of generated candidates

Standard generative-model quality metrics were computed for 1, 000 candidates at each target (Table 9). Validity is the fraction of candidates whose 25 physical device parameters all lie within the simulated design ranges; because the rank-based quantile inverse maps candidates back inside the empirical parameter bounds by construction, every generated device is realizable within the simulated design space. Uniqueness (no duplicates at 10^−3^ resolution in normalized space) is 100% at every target. Novelty is quantified by the nearest-neighbor (NN) distance to the training set in the normalized feature space: no candidate is a copy of a training device, and the fraction of candidates lying farther from their nearest training neighbor than the median training-internal NN distance rises from 93.7% at the median target to 100% at the Ultra target, with the mean NN distance growing monotonically (5.3 → 10.0, vs. a training-internal median of 3.4). The generator therefore explores progressively newer regions of the design space as the target moves beyond the empirical distribution, rather than reproducing training-set patterns.

**Table 9:** Generative-model quality metrics (1, 000 candidates per target).

Target	Validity	Uniqueness	Novelty (non-copy)	Novelty (beyond train NN)	Mean NN dist.
Median	100%	100%	100%	93.7%	5.28
High-PCE (90th pct)	100%	100%	100%	99.6%	7.32
Top-tier (99th pct)	100%	100%	100%	99.8%	8.56
Ultra (101 % of top)	100%	100%	100%	100%	10.03

NN: nearest neighbor in normalized feature space; training-internal median NN distance = 3.43.

### 4.14 Representative generated device candidates

**Figure 24:**
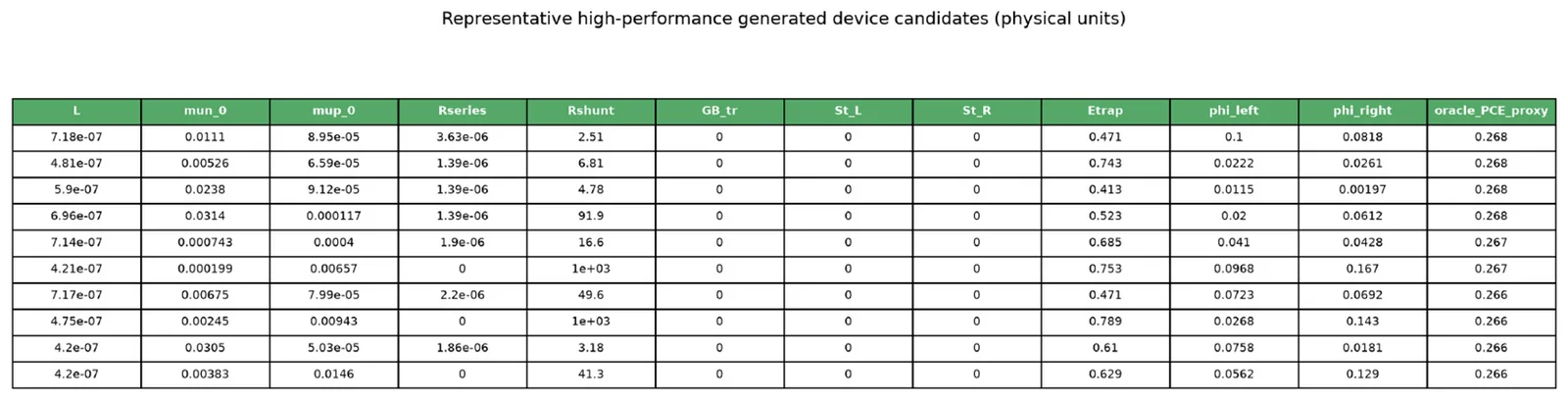
Top-10 generated device candidates at the Top-tier target, decoded to physical units, with oracle-predicted PCE proxy.

Figure 24 presents the ten highest-ranked generated devices at the Top-tier target, decoded back to physical units. The recipes are physically transparent and consistent with the explainability analysis: every top candidate is generated with vanishing grain-boundary and interfacial trap densities (GB_tr = St_L = St_R = 0), series resistance at the low edge of the simulated range (≲4 × 10^−6^Ωm^2^), healthy shunt resistance, absorber thickness in the 420–720nm band, and strong right-interface doping (10^22^–10^24^m^−3^)—i.e., the generator independently rediscovers the trap-free, low-series-resistance, well-doped design corner that device physics identifies as optimal.

### 4.15 Final result-analysis summary

**Figure 25:**
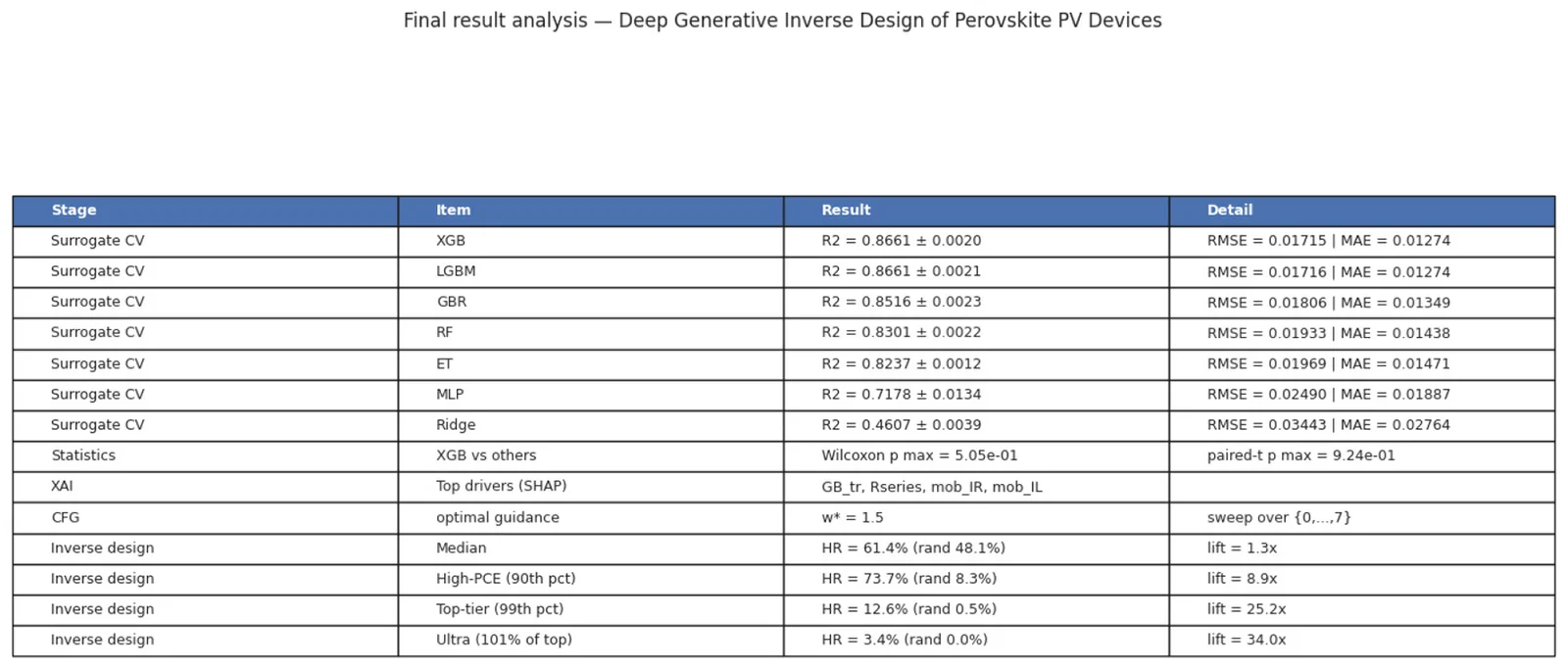
Final result-analysis table consolidating forward-surrogate metrics, pairwise statistical-test outcomes, and inverse-design hit-rates for the proposed framework.

A consolidated summary of all quantitative findings is presented in Figure 25, integrating the forward-surrogate ranking, the statistical-test outcomes, and the inverse-design hit-rates.

## 5 Conclusion

This work has presented a complete, reproducible, and statistically rigorous framework for AI-guided inverse design of perovskite photovoltaic devices. The framework integrates physics-informed feature engineering, an ensemble of forward surrogate models trained under strict leakage-free five-fold cross-validation, a multi-method explainable-AI analysis, and a conditional variational autoencoder with FiLM and classifier-free guidance.

Three principal findings are established. First, under a strictly leakage-controlled protocol in which every pre-processing estimator is refitted inside each cross-validation fold, an XGBoost forward surrogate attains an out-of-fold *R*^2^ = 0.8661 ± 0.0020 on a unified PCE proxy, statistically outperforming five competing regressors under both Wilcoxon signed-rank and paired-*t* tests while remaining statistically indistinguishable from LightGBM; effect-size analysis distinguishes the practically meaningful margins (over linear and neural baselines) from the practically negligible ones (among gradient-boosting variants). Second, the explainable-AI analysis converges on parasitic series resistance and grain-boundary defect density as the dominant efficiency-limiting parameters globally, and the class-resolved analysis shows that series resistance is mechanism-independent whereas trap-related rankings are mechanism-specific—reproducing well-established device-physics intuition and providing actionable, regime-aware experimental guidance. Third, the proposed cVAE+CFG framework, at a guidance optimum (w^*^ = 1.5) that is stable across random seeds, data splits and targets, multiplies tail access over random sampling by 8.9 × at the 90th-percentile target, 25.2 × at the 99th-percentile target and ≥34 × at an extrapolative target beyond the 99th-percentile specification, while generating exclusively valid, unique and predominantly novel device candidates whose highest-ranked recipes independently rediscover the trap-free, low-series-resistance design corner.

Several limitations should be acknowledged. The performance metric is a PCE proxy derived from the drift-diffusion outputs rather than a directly re-simulated certified efficiency, and all reported hit-rates are certified by a learned oracle ensemble whose own error is unquantified in the extrapolative regime; the absolute tail hit-rates should therefore be interpreted as oracle-certified pending re-simulation, for which the top-ranked candidates are provided in simulator-ready physical units. The underlying steady-state simulator excludes ion migration, hysteresis and degradation kinetics, so the generated recipes optimize the steady-state device physics only. The dataset represents a single absorber composition and a fixed device architecture; transferability to other absorber compositions, transport-layer stacks, or tandem architectures remains to be established, and domain-adaptation or transfer-learning strategies (e.g., fine-tuning the FiLM conditioning pathway on a small simulation corpus of the new architecture) are a natural mechanism for such extension. Finally, this work benchmarks guided generation against its own unguided ablation and random sampling; systematic comparison against alternative inverse-design families—denoising-diffusion models, normalizing flows, Bayesian optimization and reinforcement-learning-assisted search—under an identical oracle-validation protocol is an important direction that the released implementation is designed to facilitate.

Future work will pursue four directions: (i) re-simulation of generated candidates within the underlying drift-diffusion solver to close the active-learning loop with ground-truth verification of the oracle-certified hit-rates; (ii) extension of the conditioning vector to include stability-related quantities (e.g., accelerated-aging descriptors or temperature coefficients), enabling multi-objective inverse design over efficiency and operational lifetime; (iii) transfer of the framework to denoising-diffusion and consistency models on tabular data; and (iv) joint design across compositional and device-architectural descriptors in a unified generative model. Experimental validation of the most promising generated candidates through targeted fabrication and characterization is the essential next step in translating the inverse-design framework from *in silico* demonstration to laboratory impact.

## Data Availability

The dataset analyzed for this study can be found in the Kaggle repository and the complete implementation is publicly released at https://github.com/mrwick4468/perovskite-inverse-design.

## References

[B1] Biswas B. C. Islam A. T. M. S. Shimul A. I. Abedin S. M. Alsuhaibani A. M. Refat M. S. . (2026a). Integrated machine learning and impedance spectroscopy analysis of bifacial lead-free Rb_2_LiTlBr_6_ double perovskite solar cells using numerical simulation. Polyhedron 293, 118136. doi: 10.1016/j.poly.2026.118136

[B2] Biswas B. C. Shimul A. I. Paul I. AlFaify S. Benghanem M. Rahman M. A. . (2026b). Investigating the optoelectronic properties and photovoltaic performance of Na_2_AuGaBr_6_ based double perovskite solar cells via numerical simulation and AI techniques. Sci. Rep. 16:11218. doi: 10.1038/s41598-026-41519-x 41760704 PMC13046740

[B3] Breiman L. (2001). Random forests. Mach. Learn. 45, 5–32. doi: 10.1023/A:1010933404324

[B4] Burgelman M. Nollet P. Degrave S. (2000). Modelling polycrystalline semiconductor solar cells. Thin Solid Films 361, 527–532. doi: 10.1016/S0040-6090(99)00825-1

[B5] Castelli I. E. Olsen T. Datta S. Landis D. D. Dahl S. Thygesen K. S. . (2012). Computational screening of perovskite metal oxides for optimal solar light capture. Energy Environ. Sci. 5, 5814–5819. doi: 10.1039/C1EE02717D

[B6] Chandra Biswas B. Kriaa K. Shimul A. I. Maatki C. Rahman M. A. Elboughdiri N. (2025). Deep insights into lead-free Sr_3_BiI_3_-based anti-perovskite solar cells: optimization strategies and impedance spectroscopy via numerical simulation and machine learning. RSC Adv. 15, 43702–43726. doi: 10.1039/D5RA07289A 41229957 PMC12604019

[B7] Chen C. Ye W. Zuo Y. Zheng C. Ong S. P. (2019). Graph networks as a universal machine learning framework for molecules and crystals. Chem. Mater. 31, 3564–3572. doi: 10.1021/acs.chemmater.9b01294

[B8] Chen T. Guestrin C. (2016). “XGBoost: a scalable tree boosting system,” in Proceedings of the 22nd ACM SIGKDD international conference on knowledge discovery and data mining, 785–794. doi: 10.1145/2939672.2939785

[B9] Goodfellow I. J. Pouget-Abadie J. Mirza M. Xu B. Warde-Farley D. Ozair S. . (2014). “Generative adversarial nets,” in Advances in neural information processing systems (NeurIPS).

[B10] Grinsztajn L. Oyallon E. Varoquaux G. (2022). “Why do tree-based models still outperform deep learning on typical tabular data?,” in Advances in neural information processing systems (NeurIPS) datasets and benchmarks track. doi: 10.52202/068431-0037

[B11] Ho J. Jain A. Abbeel P. (2020). “Denoising diffusion probabilistic models,” in Advances in neural information processing systems (NeurIPS).

[B12] Ho J. Salimans T. (2022). “Classifier-free diffusion guidance,” in NeurIPS 2021 workshop on deep generative models and downstream applications.

[B13] Jacobsson T. J. Hultqvist A. García-Fernández A. Anand A. Al-Ashouri A. Hagfeldt A. . (2022). An open-access database and analysis tool for perovskite solar cells based on the fair data principles. Nature Energy 7, 107–115. doi: 10.1038/s41560-021-00941-3

[B14] Jain M. Bengio E. Hernandez-Garcia A. Liu C. H. Hernandez-Garcia A. Bengio Y. (2023). Gflownets for AI-driven scientific discovery. Dig. Disc. 3, 557–577. doi: 10.1039/D3DD00002H

[B15] Kapoor S. Narayanan A. (2023). Leakage and the reproducibility crisis in machine-learning-based science. Patterns 4:100804. doi: 10.1016/j.patter.2023.100804 37720327 PMC10499856

[B16] Karras T. Aittala M. Lehtinen J. Hellsten J. Aila T. Laine S. (2024). “Guiding a diffusion model with a bad version of itself,” in Advances in neural information processing systems (NeurIPS). doi: 10.52202/079017-1679

[B17] Ke G. Meng Q. Finley T. Wang T. Chen W. Ma W. . (2017). “LightGBM: a highly efficient gradient boosting decision tree,” in Advances in neural information processing systems (NeurIPS).

[B18] Kingma D. P. Welling M. (2014). “Auto-encoding variational Bayes,” in International conference on learning representations (ICLR).

[B19] Kraskov A. Stögbauer H. Grassberger P. (2004). Estimating mutual information. Phys. Rev. E 69:066138. doi: 10.1103/PhysRevE.69.066138 15244698

[B20] Le Corre V. M. Sherkar T. S. Koopmans M. Koster L. J. A. (2021). Identification of the dominant recombination process for perovskite solar cells based on machine learning. Cell Rep. Phys. Sci. 2:100598. doi: 10.1016/j.xcrp.2021.100346

[B21] Liu Y. Tan X. Liang J. Han H. Xiang P. Yan W. (2023). Machine learning for perovskite solar cells and component materials: key technologies and prospects. Adv. Funct. Mater. 33:2214271. doi: 10.1002/adfm.202214271

[B22] Loshchilov I. Hutter F. (2019). “Decoupled weight decay regularization,” in International conference on learning representations (ICLR).

[B23] Lundberg S. M. Lee S.-I. (2017). “A unified approach to interpreting model predictions,” in Advances in neural information processing systems (NeurIPS), 4765–4774.

[B24] McElfresh D. Khandagale S. Valverde J. . (2023). “When do neural nets outperform boosted trees on tabular data?,” in Advances in neural information processing systems (NeurIPS). doi: 10.52202/075280-3337

[B25] National Renewable Energy Laboratory (2025). Best research-cell efficiency chart. Available online at: https://www.nrel.gov/pv/cell-efficiency.html (Accessed 2025).

[B26] Perez E. Strub F. de Vries H. Dumoulin V. Courville A. (2018). “FiLM: visual reasoning with a general conditioning layer,” in Proceedings of the AAAI conference on artificial intelligence. doi: 10.1609/aaai.v32i1.11671

[B27] Pilania G. Mannodi-Kanakkithodi A. Uberuaga B. P. Ramprasad R. Gubernatis J. E. Lookman T. (2016). Machine learning bandgaps of double perovskites. Sci. Rep. 6:19375. doi: 10.1038/srep19375 26783247 PMC4726030

[B28] Sarker A. S. Shimul A. I. Tarekuzzaman M. Biswas B. C. (2026). Eco-friendly Cs_2_SnGeCl_6_ perovskite absorber: a combined numerical simulation and machine learning analysis for high efficiency solar cells. Mater. Today Electr. 16:100208. doi: 10.1016/j.mtelec.2026.100208

[B29] Shimul A. I. Chandra Biswas B. Ghosh A. Alrafai H. A. Hassan A. A. (2025). A study on optoelectronic properties and charge transport layer influence in novel Sr_3_PF_3_-based perovskite solar cells using numerical simulation and machine learning. Solar Energy Mater. Solar Cells 293:113838. doi: 10.1016/j.solmat.2025.113838

[B30] Shockley W. Queisser H. J. (1961). Detailed balance limit of efficiency of *p*-*n* junction solar cells. J. Appl. Phys. 32, 510–519. doi: 10.1063/1.1736034

[B31] Stranks S. D. (2017). Nonradiative losses in metal halide perovskites. ACS Energy Lett. 2, 1515–1525. doi: 10.1021/acsenergylett.7b00239

[B32] Wilcoxon F. (1945). Individual comparisons by ranking methods. Biometr. Bull. 1, 80–83. doi: 10.2307/3001968

[B33] Xie T. Fu X. Ganea O.-E. Barzilay R. Jaakkola T. (2022). “Crystal diffusion variational autoencoder for periodic material generation,” in International conference on learning representations (ICLR).

[B34] Xie T. Grossman J. C. (2018). Crystal graph convolutional neural networks for an accurate and interpretable prediction of material properties. Phys. Rev. Lett. 120:145301. doi: 10.1103/PhysRevLett.120.145301 29694125

[B35] Yang Z. Yorke S. K. Knowles T. P. J. Buehler M. J. (2025). Learning the rules of peptide self-assembly through data mining with large language models. Sci. Adv. 11:eadv1971. doi: 10.1126/sciadv.adv1971 40138415 PMC11939049

